# Inferring Demographic History from a Spectrum of Shared Haplotype Lengths

**DOI:** 10.1371/journal.pgen.1003521

**Published:** 2013-06-06

**Authors:** Kelley Harris, Rasmus Nielsen

**Affiliations:** 1 Department of Mathematics, University of California Berkeley, Berkeley, California, United States of America; 2 Department of Integrative Biology, University of California Berkeley, Berkeley, California, United States of America; 3 Department of Statistics, University of California Berkeley, Berkeley, California, United States of America; 4 Center for Bioinformatics, University of Copenhagen, Copenhagen, Denmark; Ecole Polytechnique Federale de Lausanne, Switzerland

## Abstract

There has been much recent excitement about the use of genetics to elucidate ancestral history and demography. Whole genome data from humans and other species are revealing complex stories of divergence and admixture that were left undiscovered by previous smaller data sets. A central challenge is to estimate the timing of past admixture and divergence events, for example the time at which Neanderthals exchanged genetic material with humans and the time at which modern humans left Africa. Here, we present a method for using sequence data to jointly estimate the timing and magnitude of past admixture events, along with population divergence times and changes in effective population size. We infer demography from a collection of pairwise sequence alignments by summarizing their length distribution of tracts of identity by state (IBS) and maximizing an analytic composite likelihood derived from a Markovian coalescent approximation. Recent gene flow between populations leaves behind long tracts of identity by descent (IBD), and these tracts give our method power by influencing the distribution of shared IBS tracts. In simulated data, we accurately infer the timing and strength of admixture events, population size changes, and divergence times over a variety of ancient and recent time scales. Using the same technique, we analyze deeply sequenced trio parents from the 1000 Genomes project. The data show evidence of extensive gene flow between Africa and Europe after the time of divergence as well as substructure and gene flow among ancestral hominids. In particular, we infer that recent African-European gene flow and ancient ghost admixture into Europe are both necessary to explain the spectrum of IBS sharing in the trios, rejecting simpler models that contain less population structure.

## Author Summary

In this paper, we study the length distribution of tracts of identity by state (IBS), which are the gaps between pairwise differences in an alignment of two DNA sequences. These tract lengths contain information about the amount of genetic diversity that existed at various times in the history of a species and can therefore be used to estimate past population sizes. IBS tracts shared between DNA sequences from different populations also contain information about population divergence and past gene flow. By looking at IBS tracts shared within Africans and Europeans, as well as between the two groups, we infer that the two groups diverged in a complex way over more than 40,000 years, exchanging DNA as recently as 12,000 years ago. Besides having anthropological importance, the history we infer predicts the distribution of pairwise differences between humans extremely accurately at a fine-scale level, which may aid future scans for natural selection in the genome. Despite our current focus on human data, the method is general enough to use on other organisms and has the potential to shed light on the demography of many more species.

## Introduction

Over the past several decades, population genetics has made key contributions to our understanding of human demography, as well as the demographic history of other species. Early studies that inferred haplotype trees of mitochondria and the Y chromosome [1], [2] changed our view of human origins by prompting wide acceptance of the out of Africa replacement hypothesis. Equally important were early methods that modeled the distribution of pairwise differences [3], [4] and polymorphic sites [5] in genetic samples, using this information to estimate historical population sizes and detect recent population growth. These methods revealed that a population bottleneck accompanied the human migration out of Africa; they have also shed light on recent population growth brought on by agriculture.

Advances in computational statistics have gradually made it possible to test more detailed hypotheses about demography. One advancement has been computing the coalescent likelihood of one or a few markers sampled across many organisms [6]–[11]. With the availability of likelihood methods, complex models including both gene flow and population divergence [12], and/or involving multiple populations can be analyzed. Unfortunately, full likelihood methods are not applicable to genome-scale datasets because of two significant limitations: 1) they do not scale well in the number of loci being analyzed and 2) they are not well suited for handling recombination. Methods by Yang and Rannala, Gronau, *et al.*, and Nielsen and Wakeley, among others [12]–[14], integrate over explicitly represented coalescence trees to find the joint likelihoods of short loci sampled from far apart in the genome, assuming that recombination is absent within each locus and that different loci are unlinked. The second assumption is realistic if loci are sampled far apart, but the first is problematic given that mutation and recombination rates are the same order of magnitude in humans and many other species. Simulation studies have shown that neglecting intra-locus recombination can generate significant biases when inferring population sizes and divergence times by maximum likelihood [15]–[16].

A parallel advancement to likelihood methods has been the production of genome-scale datasets. These datasets provide enough signal to test demographic questions of significant interest that cannot be answered using data from a small number of loci. Genome-wide data were instrumental, for example, in unearthing the presence of Neanderthal ancestry in modern humans [17] and the antiquity of the Aboriginal Australian population [18].

Motivated by the limitations of full likelihood methods and the power of large datasets, there is great interest in developing scalable approximate methods for population genetic inference across many recombining loci. One popular strategy is approximate Bayesian computation (ABC) [19]–[21], where the basic idea is to simulate many datasets under parameters drawn from a prior and rejection-sample by accepting replicates that are similar to an observed dataset. Another popular strategy, which is especially useful for the analysis of large SNP sets and genome-wide sequence data, is to fit the site frequency spectrum (SFS) using a composite likelihood approach. The main approximation here is to regard every segregating site as an independent sample from an expected SFS that can be computed from coalescent simulations [22] or by numerically solving the Wright-Fisher diffusion equation [23], [24].

It is computationally easier to model the SFS as if it came from a collection of unlinked sites than to work with distributions of sequentially linked coalescence times. This strategy is statistically consistent in the limit of large amounts of data [25], [26], but entails the loss of useful linkage information. A different class of method that is able to harness linkage information for demographic inference is the coalescent HMM; examples include CoalHMM, the Pairwise Sequentially Markov Coalescent (PSMC), and the sequentially Markov conditional sampling distribution (SMCSD) [27]–[30]. Unlike the SFS-based methods and full likelihood methods, which require data from tens to hundreds of individuals, coalescent HMMs can infer demography from one or a few individuals. These methods assume that the sequence of times to most recent common ancestry (TMRCAs) in a sample is distributed like the output of a Markov process, which is almost (though not quite) true under the classical coalescent with recombination [31], [32]. They use more of the information from a DNA sample than SFS-based methods do, but at present have a more limited ability to model subdivision and size changes at the same time. The PSMC produces detailed profiles of past population size [28], but has limited ability to infer migration and subdivision; CoalHMM was recently generalized to accommodate subdivision and migration, but only in the context of the 6-parameter isolation with migration (IM) model [33], [34].

Linkage information can be especially revealing about recent demographic history and recent common ancestry. Many HMM-based methods have been devised to identify long haplotype tracts inherited *identical by descent* (IBD) from a single common ancestor without recombination [35]–[38], and downstream analyses can harness IBD blocks to infer recent demographic events [39]–[42]. Of particular interest are *migrant tracts* that were inherited IBD between individuals from different populations as a result of recent migration [42]–[44]; Gravel used migrant tracts to show that at least two migration “pulses” are needed to account for tracts admixed from Europe into African Americans [44]. In addition to migrant tracts, allele frequency correlations over long genetic distances (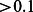 cM) have been used to study recent gene flow between European populations [45].

**Figure 1:**
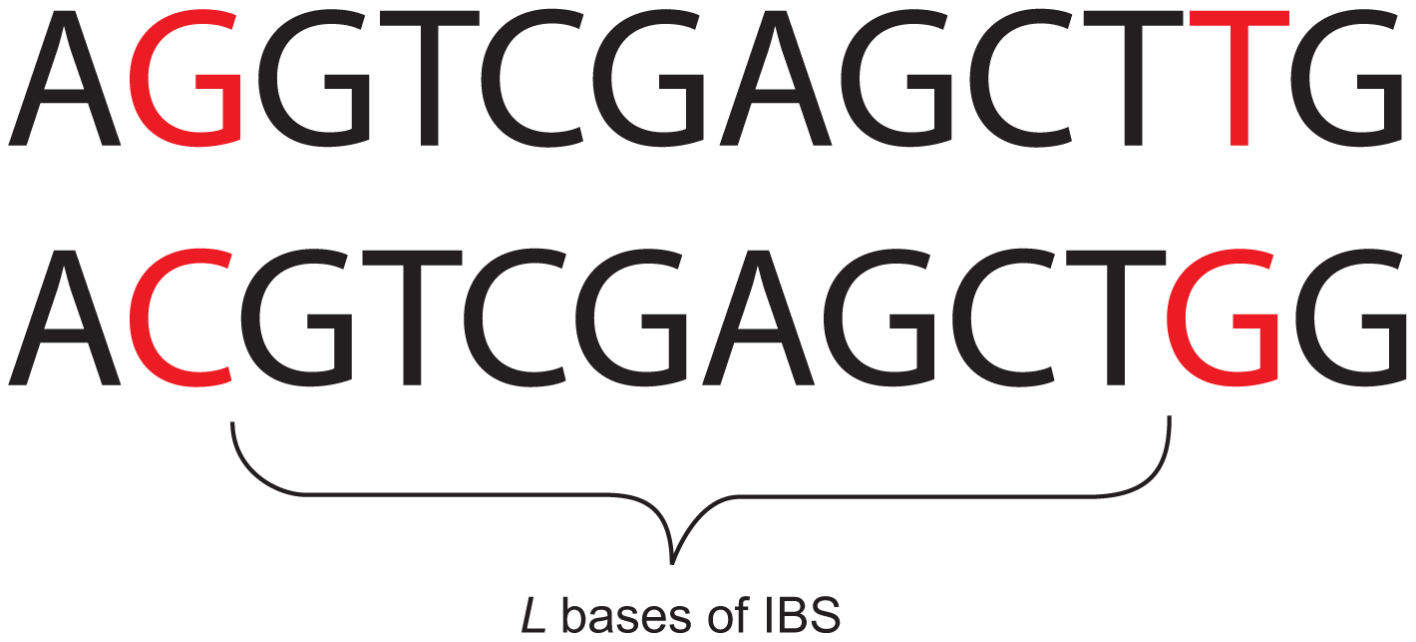
An eight base-pair tract of identity by state (IBS).

It is a challenging problem to infer recent and ancient demography within a unified theoretical framework, bridging the time gap between IBD-based accounts of recent demography and the various methods that interpret older demographic signals. To this end, we present an analytic method that draws power from linked sites over a wide range of genomic length scales, not just short blocks where linkage is strong or long sequences inherited from recent common ancestors. Specifically, we study the set of distances between neighboring SNPs in a sample of two haplotypes. The distance between adjacent polymorphisms is inversely correlated with local TMRCA; an 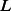-base-long locus in a pairwise alignment that coalesced 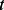 generations ago is expected to contain 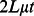 polymorphisms, 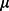 being the mutation rate per generation. This motivates us to summarize a pairwise alignment by cutting it up at its polymorphic sites and recording the length of each resulting tract of *identity by state* (IBS); for every 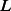, we obtain the total abundance of 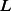-base-long IBS tracts, where an 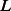-base IBS tract is defined to be 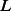 contiguous identical base pairs bracketed by SNPs on the left and right (see Figure 1).

In a non-recombining mitochondrial alignment with TMRCA 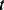, coalescent theory predicts that IBS tract lengths should be Poisson-distributed with mean 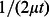. In recombining DNA, more work is required to derive the expected distribution of IBS tract lengths, but such work is rewarded by the fact that the observed spectrum is informative about a wide range of historical coalescence times. Working with McVean and Cardin's sequentially Markov coalescent (SMC) and the related SMC' model by Marjoram and Wall [32], [46], we derive an approximate closed-form formula for the expected IBS tract length distribution in a two-haplotype sample, incorporating an arbitrary number of population size changes, divergence events, and admixture pulses between diverged populations. The formula is numerically smooth and quick to compute, making it well suited to the inference of demographic parameters using a Poisson composite likelihood approach. Empirical and predicted spectra can be graphed and visually inspected in the same way that is done with the SFS, but they encode linkage information that the SFS is missing. Our source code is available for download at https://github.com/kelleyharris/Inferring-demography-from-IBS.

In simulated data, we can accurately infer the timing and extent of admixture events that occurred hundreds of generations ago, too old for migrant IBD tracts to be reliably identified and thus for the methods of Pool and Nielsen (2009), Gravel (2012), and Palamara, *et al.* (2012) to be applicable. IBS tracts have the advantage that their length distribution is directly observable; by computing this distribution under a model that incorporates intra-tract recombination, we can use the entire length spectrum for inference instead of only those short enough or long (and thus recently inherited) enough for internal recombination to be negligible. Although our derivation is for a sample size of only two haplotypes, we can parse larger datasets by subsampling all haplotype pairs and regarding them as independent. Given sufficient data, this subsampling should not bias our results, though it may reduce our power to describe the very recent past.

To illustrate the power of our method, we use it to infer a joint history of Europeans and Africans from the high coverage 1000 Genomes trio parents. Previous analyses agree that Europeans experienced an out-of-Africa bottleneck and recent population growth, but other aspects of the divergence are contested [47]. In one analysis, Li and Durbin separately estimate population histories of Europeans, Asians, and Africans and observe that the African and non-African histories begin to look different from each other about 100,000–120,000 years ago; at the same time, they argue that substantial migration between Africa and Eurasia occurred as recently as 20,000 years ago and that the out-of-Africa bottleneck occurred near the end of the migration period, about 20,000–40,000 years ago. In contrast, Gronau, *et al.* use a likelihood analysis of many short loci to infer a Eurasian-African split that is recent enough (50 kya) to coincide with the start of the out of Africa bottleneck, detecting no evidence of recent gene flow between Africans and non-Africans [14]. The older Schaffner, *et al.* demographic model contains no recent European-African gene flow either [48], but Gutenkunst, *et al.* and Gravel, *et al.* use SFS data to infer divergence times and gene flow levels that are intermediate between these two extremes [22], [49]. We aim to contribute to this discourse by using IBS tract lengths to study the same class of complex demographic models employed by Gutenkunst, *et al.* and Gronau, *et al.*, models that have only been previously used to study allele frequencies and short haplotypes that are assumed not to recombine. Our method is the first to use these models in conjunction with haplotype-sharing information similar to what is used by the PSMC and other coalescent HMMs, fitting complex, high-resolution demographic models to an equally high-resolution summary of genetic data.

## Results

### An accurate analytic IBS tract length distribution

**Figure 2:**
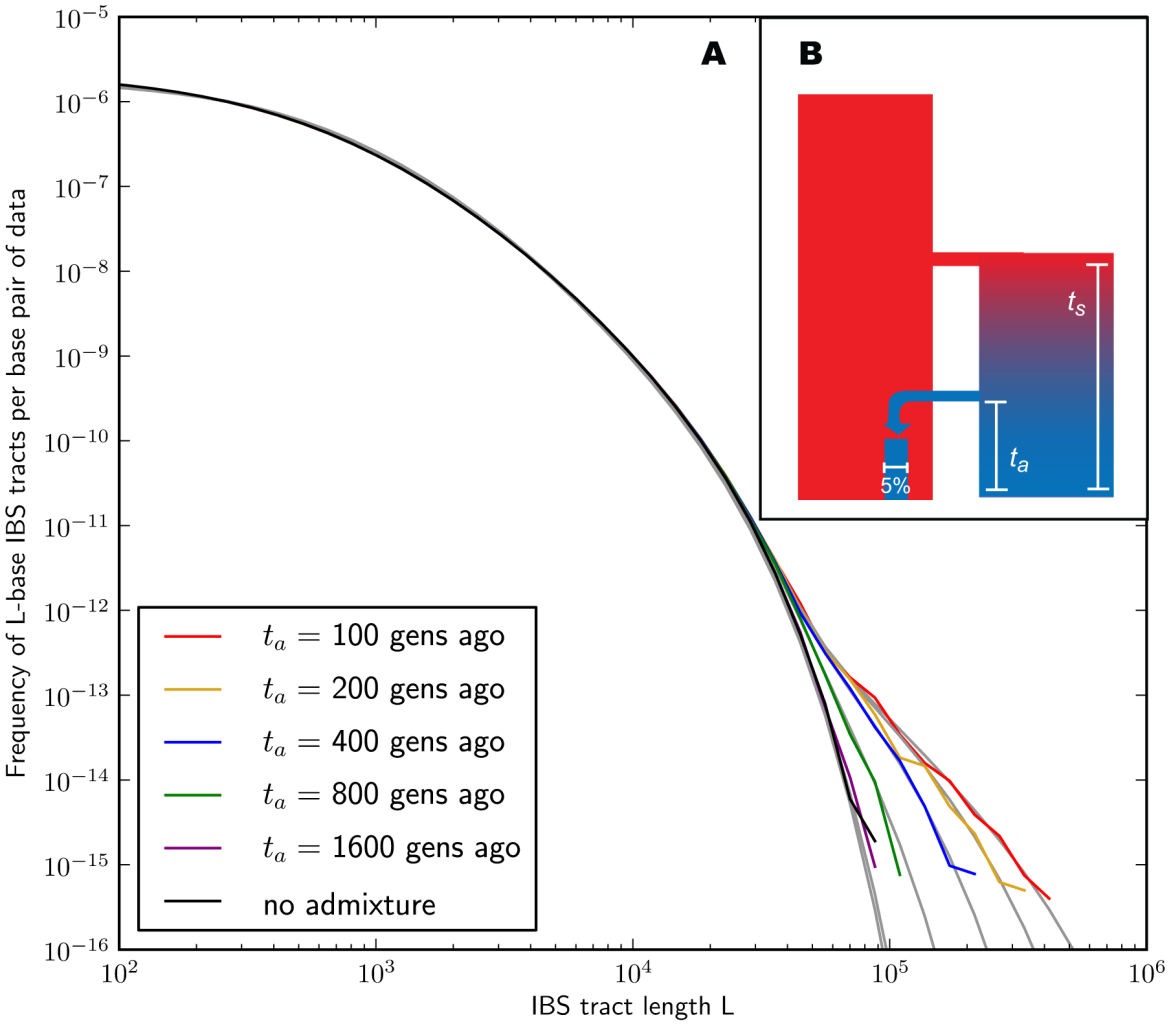
Spectra of IBS sharing between simulated populations that differ only in admixture time. Each of the colored tract spectra in Figure 2A was generated from 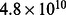 base pairs of sequence alignment simulated with Hudson's MS [68]. The IBS tracts are shared between two populations of constant size 10,000 that diverged 2,000 generations ago, with one haplotype sampled from each population. 5% of the genetic material from one population is the product of a recent admixture pulse from the other population. Figure 2B illustrates the history being simulated. When the admixture occurred less than 1,000 generations ago, it noticeably increases the abundance of long IBS tracts. The gray lines in 2A are theoretical tract abundance predictions, and fit the simulated data extremely well. To smooth out noise in the simulated data, abundances are averaged over intervals with exponentially spaced endpoints 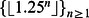.

**Figure 3:**
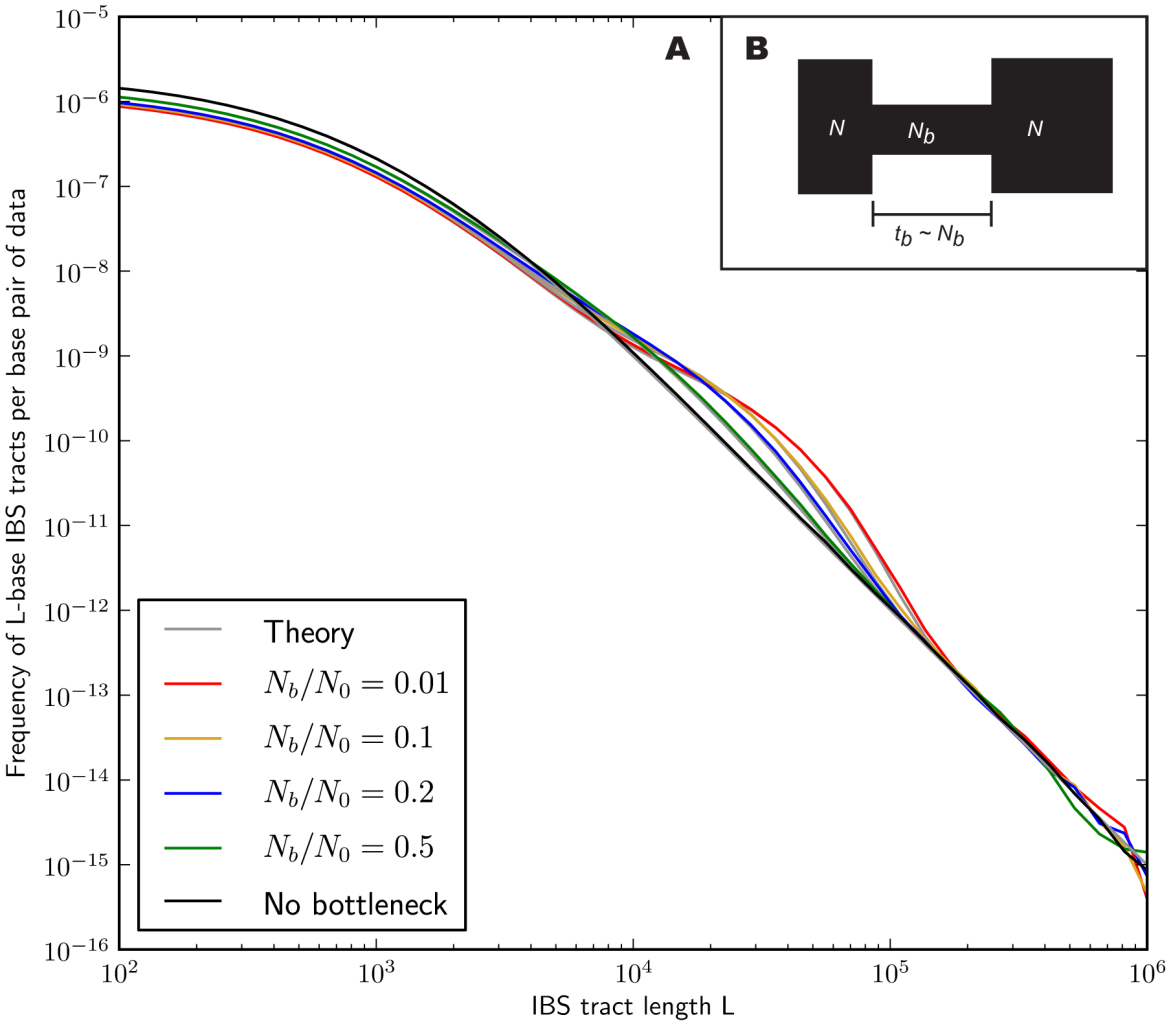
Shared IBS tracts within bottlenecked populations. As in Figure 2, each colored spectrum in Figure 3A was generated by using MS to simulate 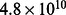 base pairs of pairwise alignment. Both sequences are derived from the population depicted in Figure 3B that underwent a bottleneck from size 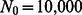 to size 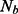, the duration of the bottleneck being 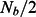 generations. 1,000 generations ago, the population recovered to size 10,000. These bottlenecks leave similar frequencies of very long and very short IBS tracts because they have identical ratios of strength to duration, but they leave different signature increases compared to the no-bottleneck history in the abundance of 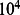
–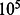-base IBS tracts. In grey are the expected IBS tract spectra that we predict analytically for each simulated history.

In the methods section, we derive a formula for the expected length distribution of IBS tracts shared between two DNA sequences from the same population, as well as the length distribution of tracts shared between sequences from diverging populations. Our formula approximates the distribution expected under the SMC' model of Marjoram and Wall [46], which in turn approximates the coalescent with recombination. We evaluate the accuracy of the approximation by simulating data under the full coalescent with recombination and comparing the results to our analytical predictions. In general, we find that the approximations are very accurate as illustrated for two example histories in Figures 2 and 3. To create each plot in Figure 2, we simulated several gigabases of pairwise alignment between populations that split apart 2,000 generations ago and experienced a 5% strength pulse of recent admixture, plotting the IBS tract spectrum of the alignment (for more details, see section 2 of Text S1). Figure 3 was generated by simulating population bottlenecks of varying duration and intensity. In both of these scenarios the analytical approximations closely follow the distributions obtained from full coalescent simulations.

If we wish to infer demography from IBS tract lengths, the following must be true: 1) IBS tract length distributions must differ significantly between data sets simulated under coalescent histories we hope to distinguish, and 2) these differences must be predictable within our theoretical framework. Figures 2 and 3 provide evidence for both of these claims. For populations that diverged 2,000 generations ago, 5% admixture is detectible if it occurred less than 1,000 generations ago, late enough for the admixed material to significantly diverge from the recipient population. Likewise, two population bottlenecks with the same strength-to-duration ratio appear distinguishable if their population sizes differ by at least a factor of two during the bottleneck. As expected, longer IBS tracts are shared between populations that exchanged DNA more recently, suggesting that IBS tracts are highly informative about past admixture times and motivating the development of a statistical demographic inference method.

### Estimates from simulated data

#### Inferring simulated population histories

Figures 2 and 3 suggest that by numerically minimizing the distance between observed and expected IBS tract spectra, we should be able to infer demographic parameters. We accomplish this by maximizing a Poisson composite likelihood function formed by multiplying the likelihoods of individual IBS tracts. Maximization is done numerically using the BFGS algorithm [50].

To assess the power and accuracy of the method, we simulated 100 replicate datasets for each of two histories with different admixture times. From each dataset, we jointly inferred four parameters: admixture time, split time, admixture fraction, and effective population size. We obtained estimates that are extremely accurate and low-variance (see Table 1); supplementary Figures S1 and S2 show the full distributions of estimated parameter values.

**Table 1:** Inferring the parameters of a simple admixture scenario.

	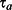 (gens)	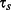 (gens)	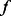	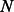
True value:	400	2,000	0.05	10,000
Mean:	431	1,990	0.0505	9,806
Std dev:	51	41	0.00652	27
Bias:	31	−10	0.0005	−194
Mean squared error:	3280	1781	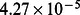	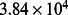
True value:	200	2,000	0.05	10,000
Mean:	220	1,983	0.0499	10,003
Std dev:	28	39	0.00328	287
Bias:	20	−17	−0.0001	−3
Mean squared error:	1184	1810	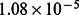	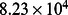

Using MS, we simulated 200 replicates of the admixture scenario depicted in Figure 2B. In 100 replicates, the gene flow occurred 400 generations ago, while in the other 100 replicates it occurred 200 generations ago. Our estimates of the four parameters 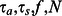 are consistently close to the true values, showing that we are able distinguish the two histories by numerically optimizing the likelihood function.

#### Comparison to 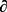
a
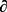
i

We compared the new method to the method implemented in 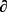
a
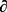
i, which can evaluate demographic scenarios with the same parameterization as ours, focusing on the simple admixture history summarized in Table 1. After simulating equal amounts of IBS tract and SFS data, we performed 20 numerical optimizations with each method starting from random points in the parameter space. Optimizations of the IBS tract likelihood were almost always successful, converging to the global optimum, but optimizations performed using default 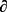
a
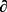
i settings often terminated near random initial starting points (see Section 4.1 of Text S1 and Table S1). This suggests that the analytic IBS-based method has greater numerical stability than the implementation of 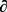
a
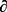
i evaluated here, at least for scenarios involving discrete admixture pulses. This is not surprising as evaluation of the likelihood function in 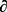
a
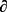
i involves the numerical solution of partial differential equations.

For a simple four-parameter history, it is feasible to identify maximum-likelihood parameters through a grid search that is robust to minor numerical instabilities. Using this type of optimization strategy, both methods provide similar results (see Supplementary Figure S3). Inspection of the likelihood surface also reveals that the two composite likelihood surfaces have different shapes–the IBS tract likelihood surface has a steeper gradient in the direction of admixture time, while the SFS likelihood changes more steeply along the divergence time axis.

### IBS tracts in human data

Our analyses of simulated data indicate that real genomic IBS tracts should contain high-resolution demographic information. A potential obstacle, especially concerning recent demography, is that random sequencing and phasing errors will tend to break up long IBS tracts. To avoid this obstacle as much as possible, we chose to study IBS sharing within the 1000 Genomes trios: one mother-father-child family who are Utah residents of central European descent (CEU) and another family recruited from the Yorubans of Ibadan, Nigeria (YRI).

**Figure 4:**
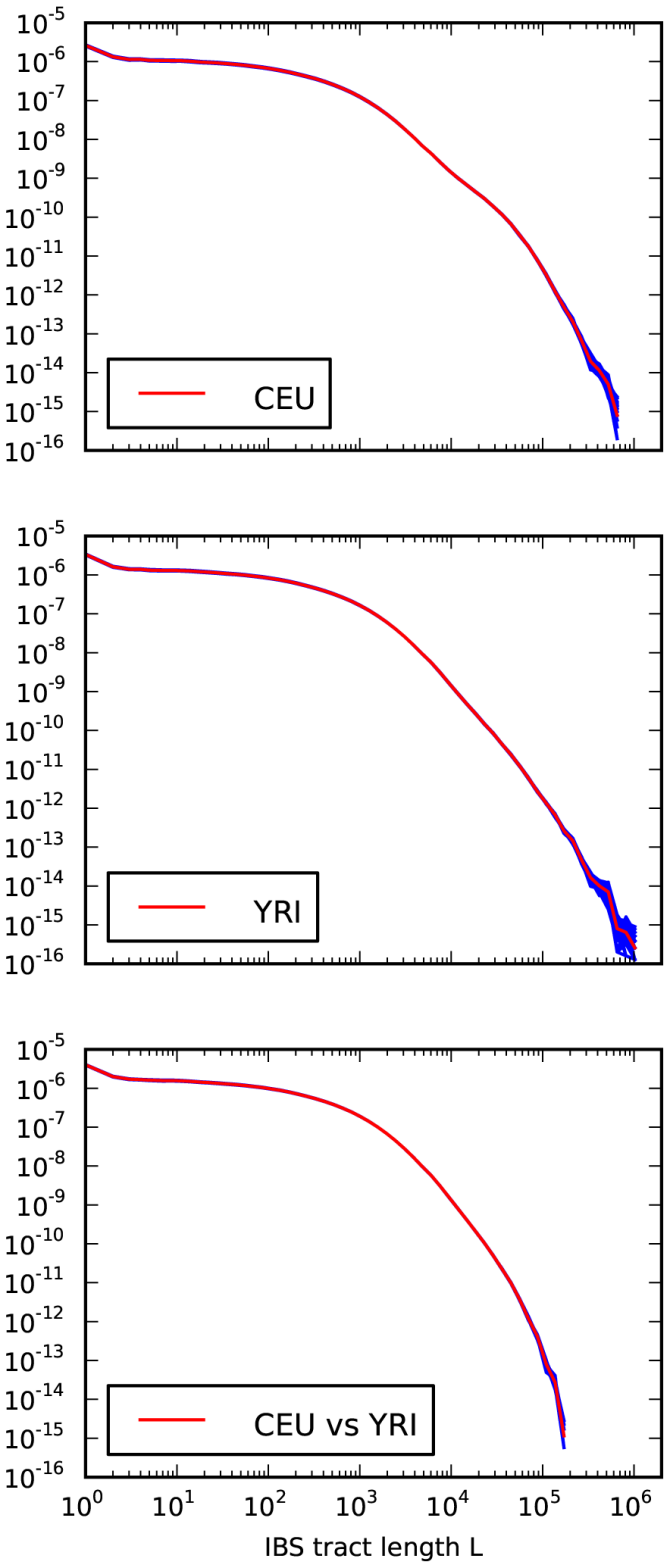
Frequencies of IBS tracts shared between the 1000 Genomes trio parental haplotypes. Each plot records the number of 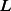-base IBS tracts observed per base pair of sequence alignment. The red spectrum records tract frequencies compiled from the entire alignment, while the blue spectra result from 100 repetitions of block bootstrap resampling. A slight upward concavity around 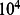 base pairs is the signature of the out of Africa bottleneck in Europeans.

We recorded the spectrum of IBS tracts shared between each pair sampled from the eight parental haplotypes, which were sequenced at 20–60x coverage and phased with the help of the children by the 1000 Genomes consortium [51]. As expected, we observe longer tracts shared within each population than between Europeans and Africans. The distribution of tracts shared between the populations, as well as within each population, were extremely robust to block bootstrap resampling (see Figure 4).

#### Sequencing and phasing errors

**Figure 5:**
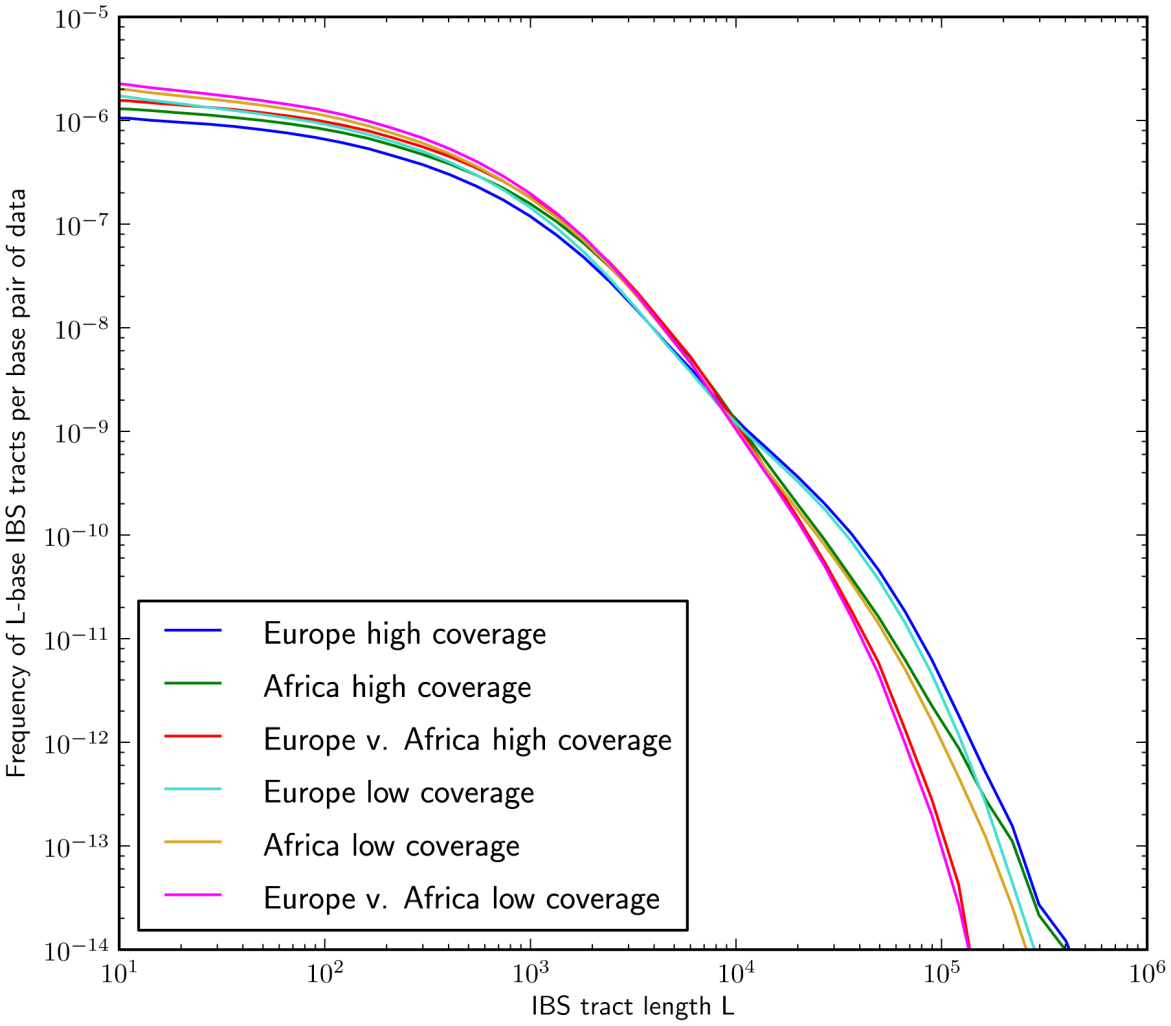
IBS tract lengths in the 1000 Genomes pilot data: trios v. low coverage. These IBS tract spectra were generated from pairwise alignments of the 1000 Genomes high coverage trio parental haplotypes and the CEU (European) and YRI (Yoruban) low coverage haplotypes, aligning samples within each population and between the two populations. Due to excess sequencing and phasing errors, the low coverage alignments have excess closely spaced SNPs and too few long shared IBS tracts. Despite this, frequencies of tracts between 1 and 100 kB are very similar between the two datasets and diagnostic of population identity.

To gauge the effects of sequencing and phasing errors on IBS tract frequencies in real data, we also generated IBS tract spectra from samples that were sequenced at 2–4x coverage from the CEU and YRI populations, also as part of the 1000 Genomes pilot project [51]. Within each population, we found that samples sequenced at low coverage shared a higher frequency of short tracts and a lower frequency of long tracts than the high coverage trio parents did. (see Figure 5). In section 3.2 of Text S1 and Figure S4, we mathematically describe how uniformly distributed errors can account for much of the difference between the high and low coverage data sets. It is encouraging that the frequencies of IBS tracts between 1 and 100 kB in length are almost the same between the two data sets, as are the frequencies of tracts shared between European and African sequences; this suggests that IBS sharing between low coverage sequences can yield reliable information about divergence times and the not-too-recent past. If we inferred demographic parameters from low coverage data without correcting for errors, however, the errors would create an upward bias in our estimates of recent population sizes.

#### Mutation and recombination rate variation

Regardless of data quality, all empirical IBS tract spectra are potentially affected by mutation and recombination rate variation [52], [53]. Our theoretical framework would make it possible to incorporate hotspots of mutation and recombination, but doing so would incur substantial computational costs when analyzing data sampled across the entire genome. We therefore made an effort to look for signatures of rate-variation bias in the real IBS tract data and to correct for such bias in the most efficient way possible.

**Figure 6:**
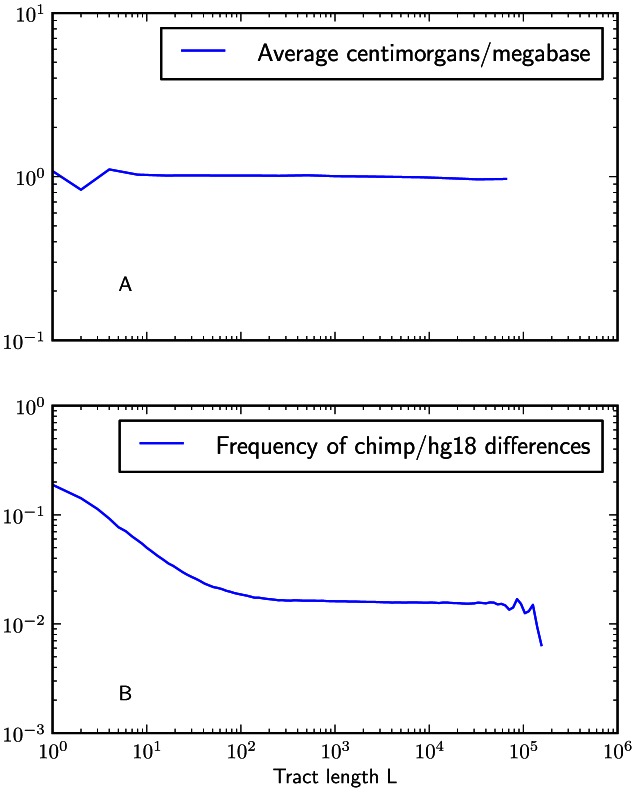
Mutation and recombination rates within 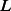-base IBS tracts. Figure 6A shows that there is no length class of IBS tracts with a significantly higher or lower mutation rate than the genome-wide average (recombination rates are taken from the deCODE genetic map [53]). In contrast, Figure 6B shows that IBS tracts shorter than 100 base pairs occur in regions with higher rates of human-chimp differences than the genomewide average. These plots were made using IBS tracts shared between Europeans and Africans, but the results are similar for IBS sharing within each of the populations.

To gauge the effects of recombination rate variation, we used the DECODE genetic map [53] to calculate the average recombination rate across all sites that are part of 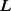-base IBS tracts. The results, plotted in Figure 6A, show no significant difference between the average recombination rate within long IBS tracts versus short ones. If recombination hotspots significantly reduced the frequency of long IBS tracts compared to what we would expect under the assumption of constant recombination rate, then the longest observed IBS tracts should span regions of lower-than-average recombination rate; conversely, if recombination hotspots significantly increased the frequency of short IBS tracts, we would expect to see short tracts concentrated in regions of higher-than-average recombination rate. We observed neither of these patterns and therefore made no special effort to correct for recombination rate variation. Li and Durbin made a similar decision with regard to the PSMC, which can accurately infer past population sizes from data with simulated recombination hotspots.

To judge whether non-uniformity of the mutation rate was biasing the IBS tract spectrum, we computed the frequency of human/chimp fixed differences within IBS tracts of length 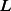. We observed that short IBS tracts of 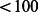 bp are concentrated in regions with elevated rates of human-chimp substitution, suggesting that mutation rate variation has a significant impact on this part of the IBS tract spectrum. IBS tracts shorter than 5 base pairs long are dispersed fairly evenly throughout the genome, but human-chimp fixed differences cover more than 10% of the sites they span (see Figure 6B) as opposed to 1% of the genome overall.

In Hodgkinson, *et al.*'s study of cryptic human mutation rate variation, they estimated that the rate of coincidence between human and chimp polymorphisms could be explained by 0.1% of sites having a mutation rate that was 33 times the mutation rate at other sites [52]. We modified our method to reflect this correction when analyzing real human data, assuming that a uniformly distributed 0.1% of sites have a scaled mutation rate of 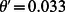, elevated above a baseline value of 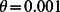. We also excluded IBS tracts shorter than 100 base pairs from all computed likelihood functions (see Methods for more detail).

### Human demography and the migration out of Africa

#### Previously published models of human demography

After generating spectra of empirical IBS tract sharing in the 1000 Genomes trios, we simulated IBS tract data under several conflicting models of human evolution that have been proposed in recent years. Two of these models were obtained from SFS data using the method 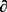
a
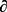
i of Gutenkunst, *et al.*; these models are identically parameterized but differ in specific parameter estimates, which were inferred from different datasets. One model was fit to the SFS of the National Institute of Environmental and Health Sciences (NIEHS) Environmental Genome Project data, a collection of 219 noncoding genic regions [24]; the other was fit by Gravel, *et al.* to a SFS of the 1000 Genomes low coverage data that was corrected for low coverage sampling bias [9]. The IBS tract length distributions corresponding to these models are qualitatively similar to each other but different from the tract length distribution of the 1000 Genomes trio data (see Supplementary Figure S5). They also differ from the tract length distribution of the 1000 Genomes low coverage data, which is much more similar to the tract length distribution of the trio data as discussed under the heading “sequencing and phasing errors.”

The models inferred from SFS data predict too few long IBS tracts shared between simulated Europeans and Africans, indicating too ancient a divergence time, too little subsequent migration, or both. There is also a dearth of long tracts shared within each population, a discrepancy that could be caused by too mild a European bottleneck and the lack of any historical size reduction in the African population.

A mild African bottleneck is a feature of the history that Li and Durbin infer using the PSMC, which also includes a more extreme European bottleneck than the ones inferred using 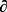
a
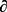
i. Compared to the 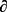
a
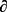
i histories, the PSMC predicts IBS tract sharing within Europe and Africa that is more similar to the pattern observed in the data (see Supplementary Figure S6), which is not surprising given that the PSMC implicitly uses IBS tract sharing for inference.

#### A new demographic model

**Figure 7:**
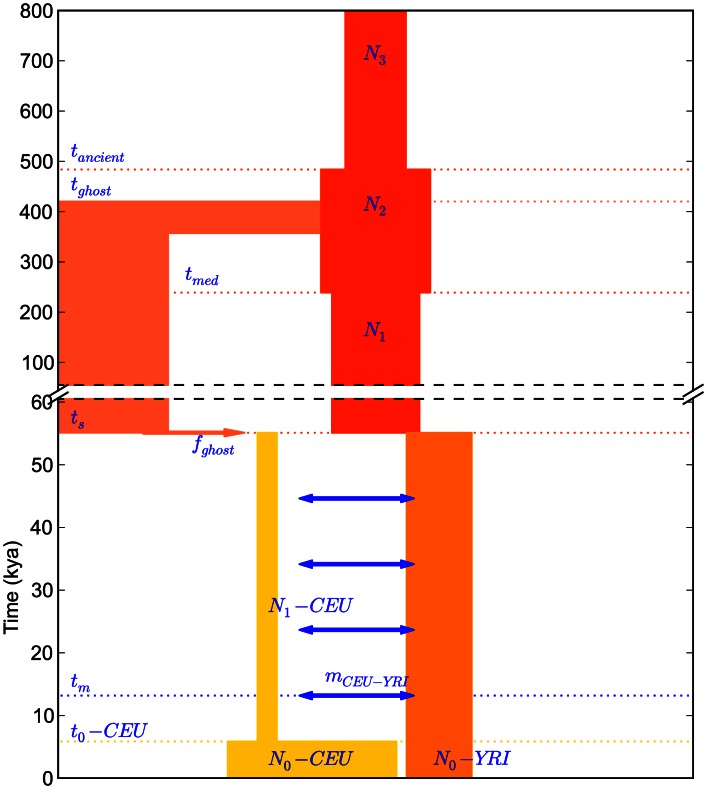
A history inferred from IBS sharing in Europeans and Yorubans. This is the simplest history we found to satisfactorily explain IBS tract sharing in the 1000 Genomes trio data. It includes ancient ancestral population size changes, an out-of-African bottleneck in Europeans, ghost admixture into Europe from an ancestral hominid, and a long period of gene flow between the diverging populations.

We were not able to match empirical IBS tract sharing in the trios by re-optimizing the parameters of a previously published history, but we were able to devise a new demographic model that is consistent with the distribution of IBS tract sharing in the trios. This model is illustrated in Figure 7. It bears many similarities to the model used by Gutenkunst, *et al.* and Gravel, *et al.*, including an ancestral population expansion, gene flow after the European-African divergence, a European bottleneck, and a recent European expansion. Unlike Gutenkunst, *et al.*, we also include a pulse of ghost admixture from an ancient hominid population into Europe, as well as a modest African population size reduction. All size changes are approximated by instantaneous events instead of gradual exponential growth.

**Figure 8:**
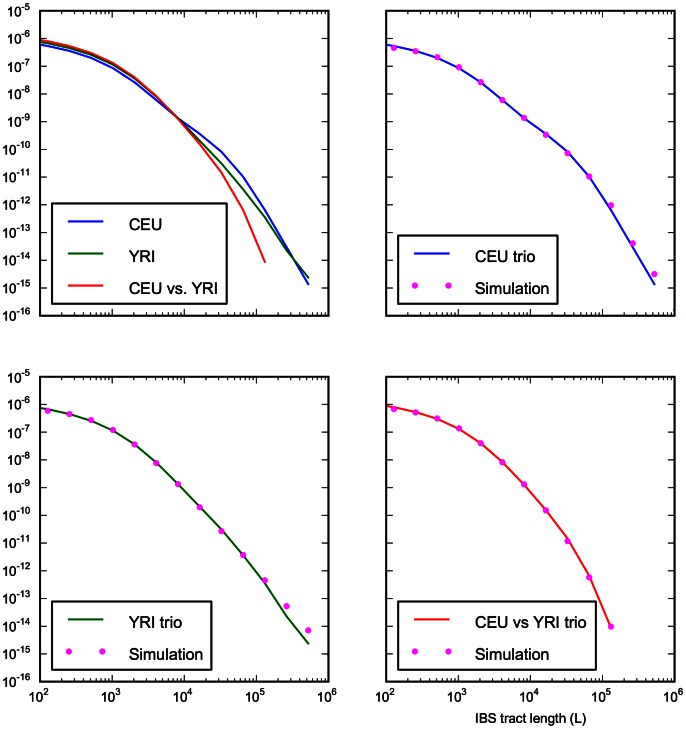
Accurate prediction of IBS sharing in the trio data. The upper left hand panel summarizes IBS tracts shared within the European and Yoruban 1000 Genomes trio parents, as well as IBS tract sharing between the two groups. The remaining three panels compare these real data to data simulated according to the history from Figure 7 with the maximum likelihood parameters from Table 2.

We fit our model to the data using a Poisson composite likelihood approach; maximum likelihood parameters are listed in Table 2. We estimate that the European-African divergence occurred 55 kya and that gene flow continued until 13 kya. About 5.8% of European genetic material is derived from a ghost population that diverged 420 kya from the ancestors of modern humans. The out-of-Africa bottleneck period, where the European effective population size is only 1,530, lasts until 5.9 kya. Given this history and parameter estimates, we simulated 12 gigabases each of European and African sequence data under the full coalescent with recombination, obtaining an IBS tract length distribution that is very close to the one observed in the trios (see Figure 8).

**Table 2:** Demographic parameters estimated from trio data.

Parameter	Estimate (kya)	Mean est. from simul.	Parameter	Estimate	Mean est. from simul.
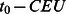	5.86	5.0	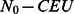	13,298	106,036
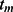	13.17	15.03	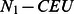	1,531	1,695
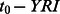	55.11	47.13	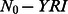	5,125	5,117
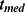	239.06	145.19	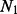	6,900	6,312
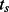	55.11	57.10	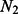	8,606	7,898
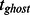	365.12	280.26	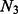	4,772	5,609
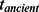	483.89	426.11	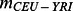	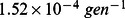	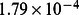
			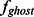	0.0589	0.0393

These times, population sizes and migration rates parameterize the history depicted in Figure 7. The migration rate 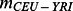 is the fraction of the European population made up of new migrants from the YRI population each generation between 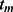 and 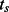; it is also the fraction of the African population made up of new European immigrants each generation during the same time period.

#### Assessing uncertainty: Block bootstrap and replicate simulations

To gauge the effects of local variation in the trio data, we re-optimized the parameters of our inferred history for each of 14 IBS tract spectra generated by block bootstrap resampling (see Figure 4). These inference results were consistent and low-variance. In addition, we used Hudson's MS to simulate 30 datasets under the inferred demographic history, then estimated demographic parameters from each simulated dataset (see Section 3.3 of Text S1 for the command line used to generate the data). This parametric bootstrapping revealed some modest parameter estimate biases, though there were no qualitative differences between the histories inferred from replicate simulations and the histories inferred from real data (see Section 3.4 of Text S1 and Figures S7, S8 and S9 for the parameter distributions inferred from simulated data). Supplementary Figure S10 compares the schematic history inferred from real data to the mean parameters inferred from simulations.

To obtain further evidence for both ghost admixture and recent migration, we inferred parameters from the trio data under two models nested within our best-fit model. For one nested model, we set the recent migration rate to zero, obtaining parameters with a significantly worse fit to the data (composite log likelihood ratio 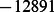 compared to the best fit model). We then simulated data under the model with no recent migration and estimated the parameters of the full model. We inferred a migration period lasting only 5 ky, the minimum length permitted by the optimization bounds.

We also considered a nested model with the ghost admixture fraction set to zero. The best model with no ghost admixture also fit significantly worse than the maximum likelihood model, with a composite log likelihood ratio of 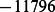. When we simulated data under the restricted model and inferred a full set of 14 parameters from the simulated data, these included a ghost admixture fraction of 0.01, the smallest fraction permitted by the optimization bounds.

Given that models inferred from site frequency spectra do not fit the IBS tracts in human data, we simulated site frequency data under our inferred demographic model to see whether the reverse was true. The resulting spectrum had more population-private alleles than the NIEHS frequency spectrum previously analyzed by Gutenkunst, *et al* (see Section 4.2 of Text S1 and Supplementary Figure S11). The discrepancy might result from biased population size estimates or from differences in the effects of errors on IBS tract and SFS data.

## Discussion

IBS tracts shared between diverging populations contain a lot of information about split times and subsequent gene flow; we can distinguish not only between instantaneous isolation and isolation with subsequent migration, but between recent admixture events that occur at modestly different times. We can accurately estimate the times of simulated admixture events that occurred hundreds of generations ago, too old for migrant tracts to be identified as IBD with tracts from a foreign population. In addition, we can distinguish short, extreme population bottlenecks from longer, less extreme ones that produce similar reductions in total genetic diversity.

Our method harnesses most of the linkage information that is utilized by Li and Durbin's PSMC and the related coalescent HMMs of Hobolth, *et al.* and Paul, Steinrücken, and Song [27], [28], [54], losing only the information about which IBS tracts are adjacent to each other in the data. In exchange for this modest information loss, our method enjoys several advantages in computational efficiency over HMMs. The runtime of an HMM is linear in the number of base pairs being analyzed, whereas we incur only a small fixed computational cost when increasing the input sequence length and/or sample size. It takes 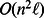 time to compute the pairwise IBS tract spectrum of 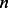 sequences that are 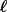 bases long, but this length distribution need only be computed once. After this is done, the time needed to find the composite likelihood of a demographic history does not depend on either 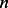 or 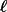. In addition, our runtime only grows linearly in the number of parameters 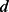 needed to describe a demographic history, whereas HMM decoding is 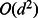. This scalability allows our program to handle all the demographic complexity that Gutenkunst, *et al.* can [24], whereas Li and Durbin are limited to a *post hoc* argument linking large or infinite population size to periods of divergence.

All parameter estimates, including admixture times, were found to be approximately unbiased in the context of a simple four-parameter model, but we observed a weak estimation bias for some parameters in the context of a complex history with 14 total parameters and very ancient demographic events. To our knowledge, no other methods have estimated such complex histories directly from the data, and we are hopeful that future improvements will help us infer complex histories more accurately. While perhaps it is disappointing that there is some bias, we emphasize that the bias is so small that it does not affect any qualitative conclusions. Two estimates that seem to be unbiased under parametric bootstrapping are the European-African divergence time of 55 kya and the date of last gene flow of 13 kya; across simulated data, we estimate a mean divergence time of 57 kya and a mean date of last gene flow of 15 kya. To minimize bias, it is crucial that we derive the IBS tract length distribution from Marjoram and Wall's SMC' [46], which provides a more accurate approximation to the correlation structure of sequential coalescence times than the earlier SMC [32] (see Methods and Supplementary Figure S12). It is possible that our method could be further improved by allowing IBS tracts to contain more than two internal recombinations; it could also be improved by allowing different parts of single tract to coalesce in epochs with different population sizes.

Our inferred human history mirrors several controversial features of the history inferred by Li and Durbin from whole genome sequence data: a post-divergence African population size reduction, a sustained period of gene flow between Europeans and Yorubans, and a “bump” period when the ancestral human population size increased and then decreased again. Unlike Li and Durbin, we do not infer that either population increased in size between 30 and 100 kya. Li and Durbin postulate that this size increase might reflect admixture between the two populations rather than a true increase in effective population size; since our method is able to model this gene flow directly, it makes sense that no size increase is necessary to fit the data. In contrast, it is possible that the size increase we infer between 240 kya and 480 kya is a signature of gene flow among ancestral hominids.

Our estimated divergence time of 55 kya is very close to estimates published by Gravel, *et al.* and Gronau, *et al.*, who use very different methods but similar estimated mutation rates to the 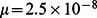 per site per generation that we use in this paper. However, recent studies of *de novo* mutation in trios have shown that the mutation rate may be closer to 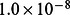 per site per generation [51], [55], [56]. We would estimate older divergence and gene flow times (perhaps 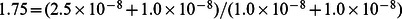 times older) if we used the lower, more recently estimated mutation rate. This is because the lengths of the longest IBS tracts shared between populations should be approximately exponentially distributed with decay rate 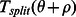.

Sustained gene flow is essential to predict the true abundance of long IBS tracts shared between the African and European populations. The inferred rate of gene flow, 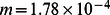 per generation, is the same order of magnitude as gene flow rates inferred from site frequency spectra using the method of Gutenkunst, *et al.* [24], [49] and by a analysis of human X chromosome diversity that employed the IM method of Hey and Nielsen [57]. The two SFS-based analyses differ from ours, however, in that global gene flow drops off at the time of the European-Asian split about 23 kya. We find that high levels of gene flow must endure past this point to explain the abundance of long IBS tracts shared between the populations in these data.

Recent gene flow is not the only form of complex population structure that has left a signature in the IBS tracts shared between Africans and Europeans–we find strong log likelihood support for a pulse of ghost admixture from an ancient hominid species into non-Africans. The admixture fraction and ghost population age are subject to some uncertainty, but our estimates of 6% and 365 kya fit the profile of admixture between non-Africans and Neanderthals that was discovered through direct comparison of ancient and modern DNA [17], [58]. Without an ancient DNA sample, we lack power to date the ghost gene flow event and assume that it occurs immediately after the European-African divergence. Sankararaman, *et al.* recently estimated that the Neanderthal gene flow event happened at least 47,000 years ago [59], much closer to estimates of the divergence date than to the present day.

To establish a less circumstantial link between Neanderthals and our inference of ghost admixture, it would be necessary to examine ancient DNA within our framework. This would be complicated by the higher error rates associated with ancient DNA sequencing and the lack of a reliable way to phase ancient samples. In general, it remains an open challenge to analyze IBS tracts shared between less pristine sequences than the ones we study here. Computational phasing programs like BEAGLE and MaCH effectively try to maximize the abundance of long IBS tracts shared between inferred haplotypes [60], [61], a fact that could seriously confound efforts to use IBS tracts for inference.

An opposite bias should result from excess sequencing errors, which have the potential to break up long shared haplotypes and degrade signals of gene flow and reduced population size. We see evidence of this degradation effect in low-coverage European and African sequences, but in the 1000 Genomes low coverage data this effect is very modest and does not noticeably influence IBS tract sharing between haplotypes from different populations. This suggests that IBS tracts in low coverage, computationally phased datasets can be used to make inferences about an intermediate-aged window of demographic history, inferences that would contribute valuable information about species where high quality data is not available and little to nothing is presently known about demography.

Even in high quality data, inference is complicated by departures of real evolutionary processes from the coalescent with uniform mutation and recombination. It is remarkable that real IBS tracts longer than 10 base pairs are distributed in a way that can be so closely approximated by our analytic predictions and by IBS tracts in simulated data; at the same time, real sequence alignments consistently harbor an excess of very short IBS tracts compared to simulated alignments, an excess we attribute to the non-uniformity of mutation rate in the genome. In this paper it was straightforward to neglect the frequencies of short tracts and correct the distribution of the remaining tracts for non-uniform mutation. In the future, however, it would be valuable to model the distribution of short tract frequencies and use them to learn more about the mutational process. At the moment, mutation rate variation is poorly understood compared to recombination rate variation, which does not appear to bias IBS tract frequencies (as seen in Figure 6). Because mutation rate variation does appear to affect IBS tract frequencies, we hope that IBS tracts can be used to obtain a more detailed picture of the mutational process just as we have used them to perform detailed inferences about demography.

Natural selection is beyond the scope of the models in this paper, but will be important for us to address in future work. One impetus for studying demography is to characterize long shared haplotypes caused by neutral events like bottlenecks so that they can be differentiated from the long shared haplotypes that hitchhike to high frequency around selected alleles [62], [63]. Histories with high SFS-based likelihoods can be quite inconsistent with genomic LD [24]; to accurately describe neutral linkage in the genome, it is essential to harness linkage information as we have done here. Schaffner, *et al.* addressed this need with their 2005 demographic model that reproduces 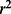 correlations between pairs of common SNPs [48], but our model explains genome-wide LD on a finer scale.

The empirical IBS tract length distributions studied here are highly similar among bootstrap subsamples, making it unlikely that they are influenced by isolated loci under strong selection or other regional peculiarities. However, the data and results could easily be influenced by background selection [64], [65]. Background selection reduces diversity in a way that has been compared to a simple reduction in effective population size [64], [66], and if selection is not being modeled explicitly, it is arguably better to report sizes that have been downwardly biased by background selection than sizes that do not accurately predict nucleotide diversity and LD.

In the future, it will be important to explain the discrepancy between the European-African site frequency spectrum studied by Gutenkunst, *et al.* and the SFS predicted by our model. The discrepancy has several potential causes, one being that the data were taken from different individuals. This could be especially important if Northern Europeans or Yorubans have significant population substructure. Another potential cause could be background selection–as previously mentioned, background selection makes coding regions look like they were generated under lower effective population size than neutral regions. We did not exclude coding regions here, opting to use as much data as possible, whereas the NIEHS frequency spectrum was recorded from a much smaller collection of intergenic loci. Bioinformatical issues may also play a role; the datasets were generated using different sequencing and filtering protocols, and even consistent bioinformatical protocols can have different effects on IBS tracts and site frequency data. A final culprit could be model specification–it is possible that a history with more structure than the one considered here could better fit the IBS tract length spectrum and the SFS simultaneously.

These caveats aside, we have here provided analytical results for the expected IBS tract length distribution within and between individuals from the same or different populations, and have shown that these results can be used to efficiently estimate demographic parameters. In the absence of likelihood-based methods for analyzing genome-wide population genetic data, methods such as the one presented here provide a computationally efficient solution to the demographic inference problem in population genetics.

## Methods

### Derivation of a frequency spectrum of shared haplotype lengths

#### A formula that is exact under the SMC

To derive an efficiently computable spectrum of shared haplotype lengths, we work within the setup of McVean and Cardin's sequentially Markov coalescent (SMC) [32] and introduce additional approximations as needed. We do not address the subject of IBS tracts in multiple sequence alignments; all alignments we refer to are pairwise.

The coalescent with recombination specifies a probability distribution on the coalescent histories that could have produced a sequence of base pairs 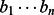. Such a history assigns a TMRCA 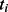 to each base pair 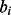, and in general the times 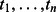 are related in a complex non-Markov way [31]. Because inference and computation under this model are so challenging, McVean and Cardin [32] introduced a simpler coalescent process (the SMC) for which
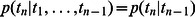
(1)and coalescences are disallowed between sequences with no overlapping ancestral material. In a population with stationary coalescence time density 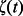 and recombination probability 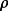 per base pair per generation, the SMC stipulates the following: If the 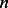
th base pair in a sequence coalesces at time 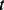, then with probability 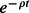 there is no recombination in the joint history of base pairs 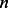 and 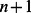 before the find a common ancestor, meaning that base pair 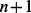 coalesces at time 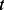 as well. With infinitesimal probability 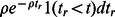, however, the joint history of the two base pairs contains a recombination at time 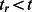. Given such a recombination, base pair 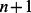 is constrained to coalesce more anciently than 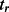. Because of the assumption of no coalescence between sequences with nonoverlapping ancestral material, the distribution of 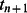 is independent of 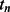 given 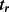. It is a renormalized tail of 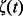:
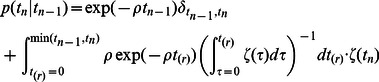
(2)


For an alignment between sequences from constant-size populations that diverged at time 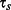, we can derive a formula for the expected IBS tract spectrum that is exact under the SMC. Specifically, we compute the expected value of 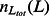, the number of 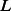-base IBS tracts in an 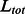-base sequence alignment. By setting 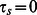, we can also compute this value for two sequences sampled within the same population.

**Figure 9:**
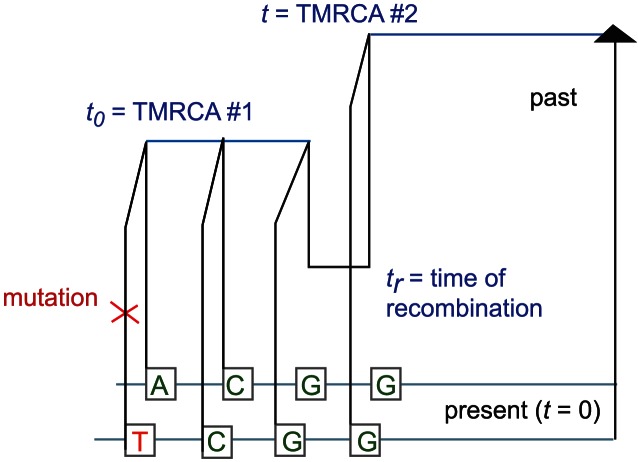
The coalescent with recombination and the sequentially Markov coalescent associate an observed pair of DNA sequences with a history that specifies a time to most recent common ancestry for each base pair. Polymorphisms are caused by mutation events, while changes in TMRCA are caused by recombination events.

In an alignment of length 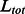, any of the leftmost 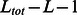 base pairs could be the leftmost polymorphic endpoint of an 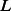-base IBS tract. Moreover, each of these 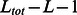 base pairs has the same *a priori* probability of being such a leftmost endpoint. This motivates us to define 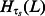 as the probability that a randomly chosen locus will be a) polymorphic and b) followed on the left by 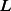 homozygous base pairs, assuming that b) is not made impossible by edge effects. Assuming uniform mutation and recombination rates 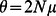 and 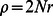, it follows that
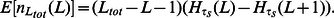
It is straightforward but computationally costly to relax the assumption of uniform mutation and recombination rates. We will wait to revisit this issue in the context of data analysis. For now, let 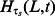 be the joint infinitesimal probability that a) a randomly selected locus 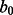 is polymorphic, b) the next 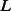 base pairs 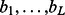 sampled from left to right are non-polymorphic, and c) the rightmost base pair 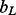 has TMRCA 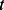. We can use the sequential Markov property of the SMC to write down a recursion for 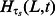 in 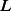: if 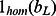 denotes an indicator function for the event that base pair 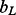 is homozygous and 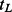 denotes the coalescence time of base pair 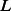, then
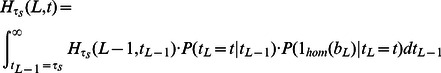
(3)

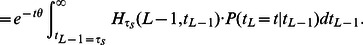
(4)When 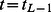, the quantity 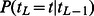 is simply 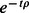, the probability that neither lineage undergoes recombination. Conversely, a recombination is required whenever 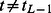; to compute 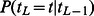 when 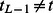, we must marginalize over the time 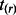 of the recombination that caused the change in TMRCA (see Figure 9). Paul, Steinrücken, and Song used a similar computation to motivate the transition probabilities of their sequentially Markov conditional sampling HMM [54]:
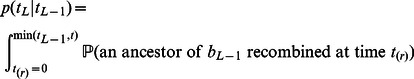
(5)

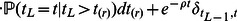
(6)

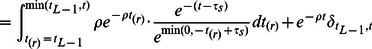
(7)

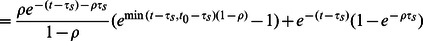


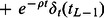
(8)


This yields that 
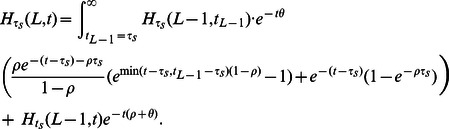
(9)


To find 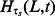, all we need to do is apply the integral operator (9) 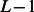 times to the base case
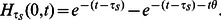
(10)Moreover, it turns out that this integral recursion can be transformed into an algebraic recursion that is more efficient to compute:

#### Claim 1

*The sampling probability* 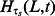 *can be written in the form*

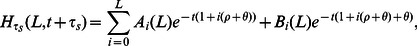
(11)
*with coefficients that satisfy the following recursions and base cases:*

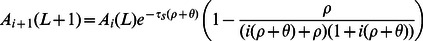
(12)

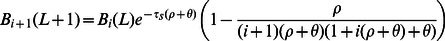
(13)

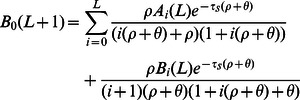
(14)

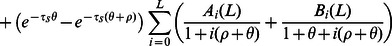


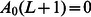
(15)

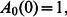
(16)

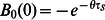
(17)


It is straightforward to prove Claim 1 by applying the integral operator (9) to expression (11). The upshot is that
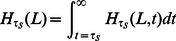
can be computed in 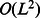 time using elementary algebra.

While Claim 1 enables an exact computation that is orders of magnitude faster than using numerical integration to solve recursion (9), it is still too slow for our purposes. It will prove more useful to derive an approximate formula for 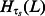 that is not exact with respect to the SMC but whose computation time does not depend on 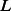; this is accomplished by limiting the total number of recombinations that can occur within the history of an IBS tract.

#### Restricting the number of ancestral recombination events

**Figure 10:**
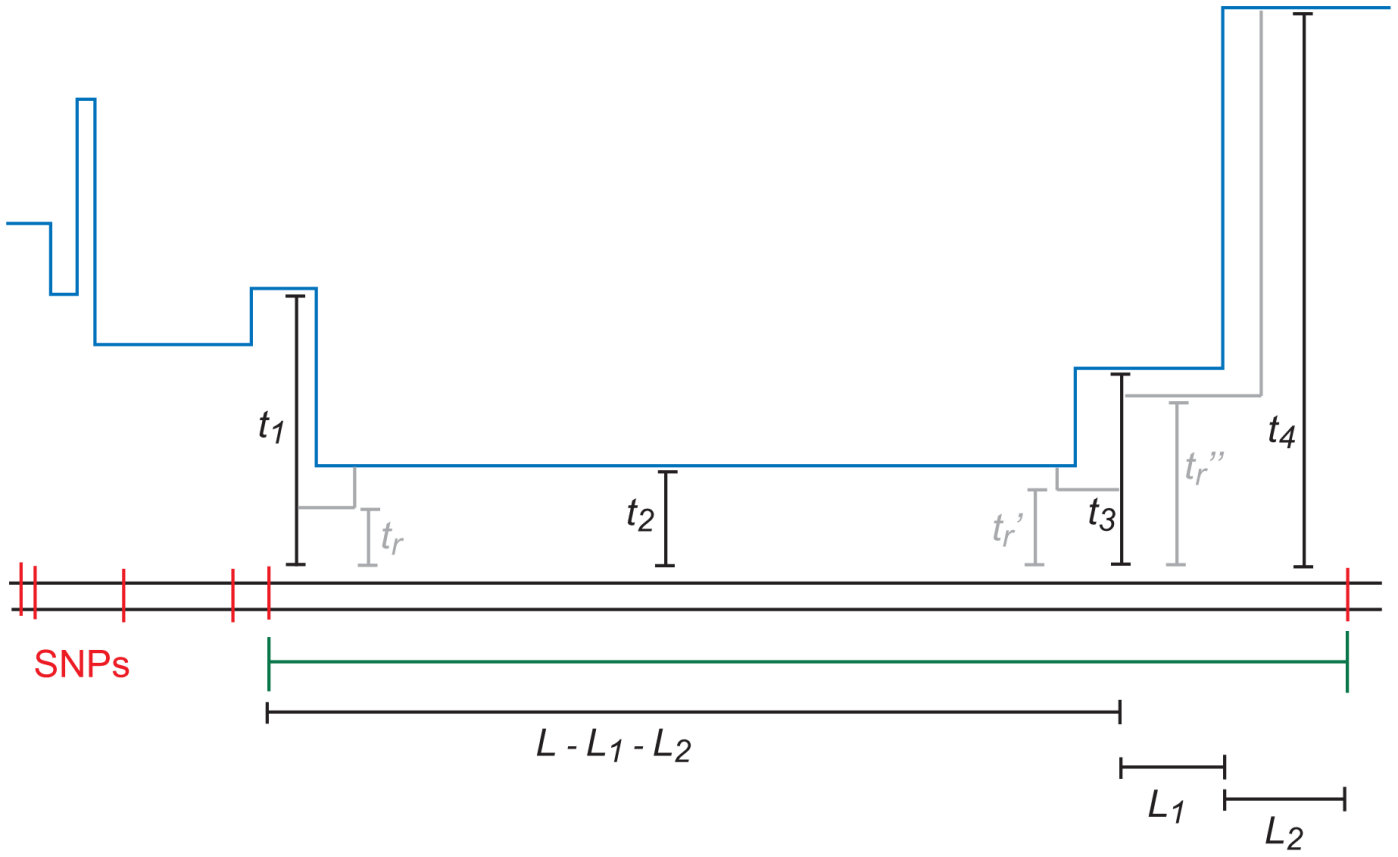
An 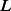-base IBS tract with three recombination events in its history. A blue skyline profile represents the hidden coalescence history of this idealized IBS tract. In order to predict the frequency of these tracts in a sequence alignment, we must integrate over the coalesence times 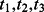 as well as the times 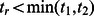, 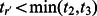, and 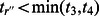 when recombinations occurred.

In principle, each base pair of an 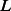-base IBS tract could coalesce at a different time, with each TMRCA partially decoupled from its neighbors by an ancestral recombination event. In practice, however, most 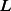-base IBS tracts will contain many fewer than 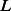 distinct TMRCAs. Figure 10 depicts an IBS tract with three distinct TMRCAs separated by 2 internal recombinations. As we move left along the history of a sequence, the probability of seeing 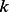 ancestral recombinations before we see a single ancestral mutation declines geometrically as 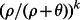. Moreover, each ancestral recombination represents a chance for the TMRCA to become ancient and force mutation to end the IBS tract soon. Lohse and Barton were able to show under the full coalescent with recombination (not the SMC) that if 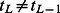, then 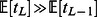 [67].

To speed the computation, we assume that an 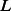-base IBS tract contains at most two internal recombinations. To make this precise, we let 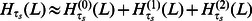, where 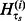 is the joint probability that a) a randomly selected base pair is polymorphic, b) the next 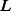 base pairs to the left are IBS, and c) the coalescent history of these 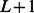 base pairs contains exactly 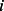 ancestral recombinations.

Computing 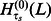 is easy because it involves integrating over only one coalescence time:
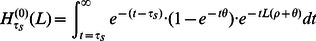
(18)

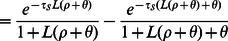
(19)


When 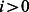, however, the complexity of the integral grows quickly. We must marginalize over 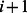 different coalescence times 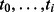, 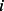 different times of recombination 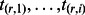, and 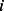 recombination breakpoint locations 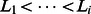. For example,
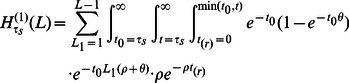
(20)

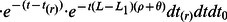
(21)In the supplementary section S??, we evaluate this expression in closed form after approximating the sum by an integral. In the same way, we compute 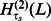 (see Section 1.2 in Text S1).

#### Adding recombination and population size changes

As demonstrated in the results section, IBS tract lengths are very informative about the timing of admixture pulses. This makes it interesting to look at IBS tracts shared between two populations A and B that diverged at time 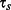 but exchanged genetic material at a more recent time 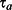. To this end, we let 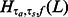 be the frequency of 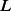-base IBS tracts shared between A and B assuming that a fraction 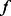 of A's genetic material was transferred over from B in a single pulse at time 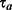, with the remaining fraction constrained to coalesce with B before 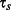. If we define 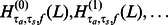 the same way as before, then 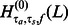 is simply a linear combination of 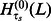 and 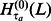:
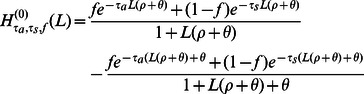
(22)

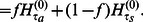
(23)The next term 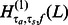 is much more challenging to compute exactly; this is done in supplementary section S1.3. The challenge stems from the fact that the recombination site might partition the tract into two components that have different “admixture statuses”– one side might be constrained to coalesce before the ancestral split time, and the other side might not (see Supplementary Figure S13). As a result 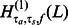 is not an exact linear combination of 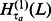 and 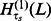.

A similar challenge arises when we consider histories where the effective population size varies with time. For a simple example, consider the vector of times 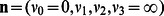 with 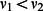 and the vector of sizes 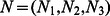. It will be useful to let 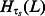 denote 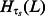 in a population where the constant effective population size is 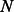. Let 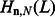 denote the frequency of 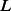-base IBS tracts in a population that underwent a bottleneck, such that the population size function 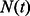 is piecewise constant with 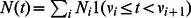. This population has a coalescence density function that is a linear combination of exponentials, which implies that 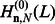 is a linear combination of the quantities 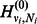:
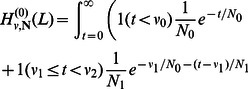
(24)

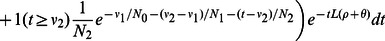


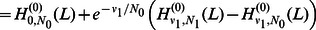
(25)

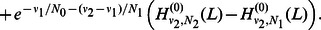



As in the case of an admixed population, the next term 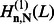 is harder to compute because it is difficult to write down the frequencies of IBS tracts that span multiple epochs (i.e. when the left hand part of a tract coalesces earlier than 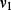 and the right hand part coalesces later than 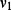 during a time period of smaller effective population size). The higher terms (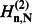, etc.) are more complicated still. Rather than attempt to compute these terms for a simple bottleneck history, we have developed an approximation for 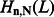 that involves little extra computation and generalizes easily to more complicated histories. The approximation can be described as the following modification to the SMC: if the left hand side of an IBS tract coalesces between 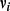 and 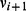 and the tract then recombines at time 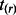, the probability distribution of the new coalescence time is 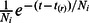 instead of 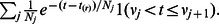 If we let 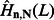 be the IBS tract spectrum under this assumption, we have that
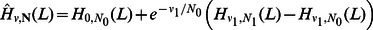
(26)

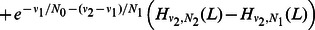
This linear approximation strategy generalizes to any history that is described by size changes, splits, and admixture pulses, since every such history has a coalescence density function that is a linear combination of exponentials. Figure 3 shows a close agreement between 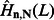 and the IBS tracts in data simulated under bottleneck histories with MS.

#### Improving accuracy via the SMC'

If we approximate the frequency of 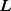-base IBS tracts by calculating 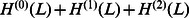 as described above, we slightly underestimate the frequency of intermediate-length tracts between 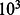 and 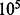 base pairs long. This underestimation can bias our estimates of population size and other demographic parameters (see Supporting Figure S22), but this bias can be substantially reduced by replacing 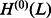, the largest summand, with a term 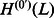 that is derived from Marjoram and Wall's SMC'.

The SMC' is a coalescent approximation that is slightly more complex and more accurate than the SMC [46]. Both the SMC and the SMC' are efficient for simulating long DNA samples with many ancestral recombinations, and both satisfy the Markov property from equation (1).

Under McVean and Cardin's SMC, 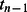 and 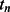 are distinct whenever a recombination occurs between 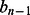 and 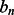. As a result, 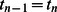 with probability 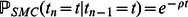. Under the SMC', the situation is more complex: in the event of an ancestral recombination between base pairs 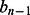 and 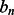, it is possible for the times 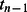 and 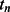 to be equal because of a “back-coalescence” involving part of the ancestral recombination graph that the SMC does not retain. In particular,
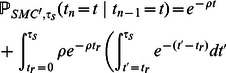
(27)

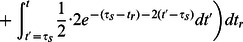
(28)

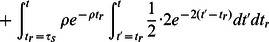
(29)

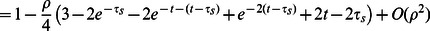
(30)


Motivated by Equation (30), we can replace 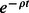 with 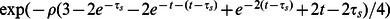 in Equation (18) to compute the probability of observing 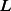 base pairs that are IBS with no internal recombinations that change the coalescence time. We obtain that
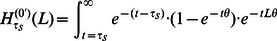
(31)

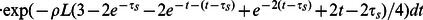
(32)

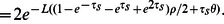
(33)

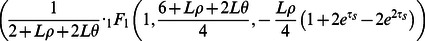
(34)

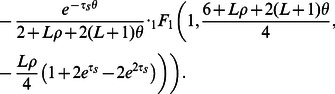
(35)In this formula,
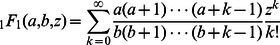
is a confluent hypergeometric function of the first kind, which we compute via the Python mpmath library.

### Inference strategy

The previous section described how we compute 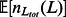, the expected number of 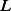-base IBS tracts present in 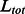 base pairs of sequence alignment. As 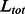 approaches infinity, the law of small numbers predicts that 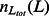 should become Poisson-distributed about its mean. This motivates us to compare models 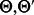 by evaluating the Poisson composite log likelihood of the IBS tract spectrum under each model:
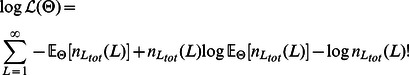
(36)


We emphasize that this is a composite likelihood function formed by multiplying likelihoods together that are not necessarily independent of each other. Nonetheless, the resulting function may provide estimators with desirable statistical properties, as illustrated in the Results section. Throughout this paper, when discussing composite likelihood functions we will use the shorter term ‘likelihood function’. However, we emphasize that we never apply general asymptotic theory for likelihood function to the composite likelihood functions derived and applied in this paper.

This formula above has a tendency to destabilize numerically; its many alternating terms must be computed by multiplying small 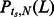 numbers by the very large number 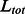, leading to a rapid loss of machine precision. This loss of precision can be avoided, however, by grouping IBS tracts into bins with endpoints 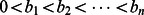 and evaluating a log likelihood function with one term per bin. In addition to improving numerical stability, binning reduces the time required to compute and optimize the likelihood function. Letting 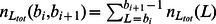, we define
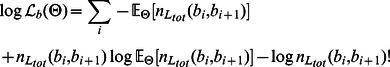
(37)


The ideal choice of bins depends on the nature of the demography being inferred. We found that exponentially spaced bins (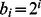) performed well for most inference purposes, and these are the bins we used to infer human demography from the 1000 Genomes trios. The optimization results were not sensitive to the fine-scale choice of binning scheme. For inferring admixture times from data simulated without population size changes, a different binning scheme was more efficient because only the longest tracts were truly informative (this is clear from looking at Figure 2). We took 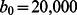 and 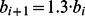.

To infer the joint history of two populations A and B, we use the quasi-Newton BFGS algorithm to simultaneously maximize the likelihood of three different IBS tract spectra: the first summarizes an alignment of two sequences from population A, the second summarizes an alignment of two sequences from population B, and the third summarizes an alignment between population A and B. The three likelihoods are computed with respect to the same set of parameters 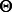 and multiplied together. Computing the joint likelihood of an 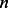-population history requires 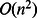 computational time compared to the likelihood of a one-population history with the same number of size change and admixture parameters.

### Mutation rate variation

The human genome is known to contain complicated patterns of mutation rate variation, as well as a better-understood map of recombination rate variation [52], [53]. As discussed in the results, only mutation rate variation appears to bias the distribution of IBS tracts and is therefore taken into account by our method. Long regions of elevated mutation rate should elevate the abundance of short IBS tracts but have little effect on the abundance of longer IBS tracts. Because the distribution of such regions is not well understood and is outside the scope of this paper, we simply restrict our inference to the spectrum of tracts longer than 100 base pairs.

Hodgkinson, *et al.*, among others, have shown that sites of elevated mutation rate are not always grouped together in the human genome [52]. They propose several models of cryptic, dispersed variation that could explain observations of correlation between human and chimp polymorphism. Of the models that they deem consistent with the data, the one that we incorporate into our method is a bimodal distribution of mutation rate where 99.9% of all sites have the baseline rate 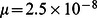 mutations per base per generation and the remaining 0.1% have an elevated rate 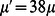. It is straightforward to compute the probability 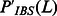 that a site of elevated mutation rate followed by 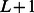 bases of normal mutation rate is the left endpoint of an 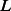-base IBS tract. If we were to randomly assign a higher mutation rate to 0.1% of the 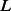 IBS bases and compute the resulting probability 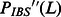, the difference between 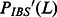 and 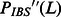 would be on the order of the miniscule difference between 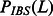 and 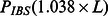. Neglecting this second effect, we replace 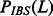 with 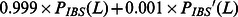 for the purpose of inferring demography from human data.

### Data analysis

For human demographic inference, we used the European and Yoruban parents who were sequenced at high coverage and phased with the help of their children by the 1000 Genomes pilot project [51]. We generated a set of IBS tract lengths from each of the six pairwise alignments between distinct CEU haplotypes, excising centromeres, telomeres, and other gaps annotated in the UCSC Genome Browser. To enable comparison of this spectrum with the spectrum of shared IBS tracts in the low coverage pilot data, we also excised regions that were inaccessible to the low coverage mapping or contained conspicuously few SNP calls in the low coverage data (see Section 3.1 of Text S1 for details). The IBS tracts shared in the remaining parts of the genome were pooled to generate a spectrum of IBS sharing within the CEU population. The same regions were used to find the IBS tract shared within the six pairwise alignments of YRI haplotypes, as well as within the 16 pairwise alignments between a CEU haplotype and YRI haplotype.

Because of our interest in comparing our method to the closely related method of Li and Durbin [28], we used the same mutation and recombination rates used in that paper (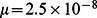 mutations per base per generation; 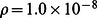 recombinations per base per generation), as well as the same generation time (25 years).

### Block bootstrapping

We performed block bootstrapping on IBS tract sharing within the CEU population by resampling large blocks, with replacement, from the 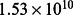 base pairs of pairwise alignment data that were obtained by matching CEU parental haplotypes with each other. We did this by partitioning the total pool of CEU-CEU sequence alignment into 100 nonoverlapping regions that were each approximately 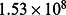 base pairs long. These regions were drawn with their boundaries at polymorphic sites so that no IBS tracts were broken up and divided between two blocks. By necessity, most blocks contain pieces of more than one continuous chromosomal region, but each is taken from a single pair of individuals. Each of the blue IBS tract length spectra from Figure 4 was created by sampling 100 blocks uniformly at random with replacement and recording the IBS tract lengths found within these blocks. The same procedure was used to sample from the distributions of tract lengths within the YRI population and between the CEU-YRI populations. Because the amount of pairwise CEU-YRI alignment totaled 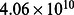 base pairs, the blocks of sequence alignment sampled from between populations were each approximately 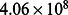 base pairs long.

## Supporting Information

Figure S1
**Each of these histograms was generated from 100 simple admixture history datasets that were simulated with gene flow time** 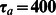 **. The true parameter value is shown in red.** All parameter estimates have low variance and appear consistent.(EPS)

Figure S2
**These histograms record the distribution of parameter estimates for 100 simple admixture histories with gene flow time** 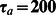 **. True parameter values are shown in red.**
(EPS)

Figure S3
**These IBS tract likelihood surfaces were generated from two of the 200 simulated data sets that were analyzed to produce** **Table 1** **in the main text, while the** 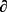 **a** 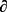 **i log likelihood surfaces were generated from an equivalent amount of simulated allele frequency data.** In each case, a grid search will accurately estimate the parameters of the simple admixture history in Figure 2 of the main text.(EPS)

Figure S4
**This figure compares IBS tract length frequencies in the 1000 Genomes low coverage pilot data to the frequencies of IBS tracts that remain in the high-coverage trio data after the addition of Poisson-distributed false SNPs to simulate an error rate of** 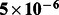 **per base pair.** In the notation of section S?? of Supplementary Text S1, it plots the low coverage IBS tract frequencies 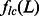 along with the error-degraded high coverage trio frequencies 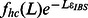. For 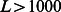, we can see that 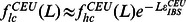, 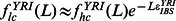, and 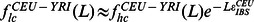 when we let 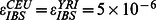.(EPS)

Figure S5 **IBS tract sharing in data simulated under the Gutenkunst,** ***et al.*** **demographic model.** Each panel here shows the length distribution of IBS tracts in the 1000 Genomes trios compared to the length distributions that are obtained by simulating data under the model of Gutenkunst, *et al.* 2009 [15]. Compared to real human data, this demographic model predicts too few long IBS tracts shared between Europeans and Africans, as well as too few long IBS tracts shared within Europe.
(EPS)

Figure S6 **IBS tract sharing in data simulated under the Li and Durbin demographic model.** Li and Durbin's PSMC does not measure the extent of gene flow between species, but implicitly uses IBS tracts to estimate past population sizes. We simulated data under each of the European and African histories published in Li and Durbin, 2011 [23] and plotted their IBS tract length frequencies against frequencies from the 1000 Genomes trios. Long tracts have similar frequencies between the real and simulated data, though short tracts are less accurately predicted by the PSMC.
(EPS)

Figure S7 **Results of block bootstrapping and parametric bootstrapping: Part I of III.** In each of these figures, the green line marks a parameter estimate obtained from the 1000 genomes trio data. Each data point contributing to the red “block bootstrap” histogram was estimated from a dataset that was created by sampling 100 bootstrap blocks from the trio data with replacement. The blue histogram records the results of inference from simulated data: each dataset was generated using the MS command line in section S?? and the maximum likelihood parameter values shown in green.
(EPS)

Figure S8 **Results of block bootstrapping and parametric bootstrapping: Part II of III.**
(EPS)

Figure S9 **Results of block bootstrapping and parametric bootstrapping: Part III of III.**
(EPS)

Figure S10 **The solid colored blocks in this figure depict the phases of the demographic history that was inferred from the 1000 genomes trio data.** The overlaid black lines depict the mean history inferred from replicate MS simulations.
(EPS)

Figure S11
**A SFS simulated under our inferred demographic history.** This model has an excess of high frequency derived alleles compared to the NIEHS data. The excess produces red off-diagonal regions in this Anscombe residual plot produced by 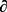
a
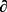
i.(EPS)

Figure S12 **Parameter inference without the SMC'.** These histograms were generated by optimizing the parameters of an IBS tract length distribution derived purely from the SMC, not the SMC'. The simulated datasets are the same ones used to generate Figure S12, but in this case, model inaccuracy leads us to underestimate the effective population size and the admixture fraction.
(EPS)

Figure S13
**This picture represents an IBS tract of mixed admixture status.** The left-hand side is admixture-negative, constrained to coalesce before the divergence time 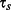, while the right-hand side is admixture positive, constrained only to coalesce before the admixture time 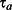.(EPS)

Table S1
**Numerical performance of** 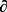 **a** 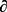 **i optimization vs. IBS tract inference.** The left table contains the results of 20 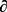
a
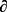
i Nelder-Mead optimizations attempting to guess demographic parameters from an allele frequency spectrum. There is no population size estimate because 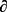
a
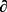
i estimates it analytically before the optimization begins. The right table contains the result of 20 analogous optimizations that use an equivalent amount of IBS tract data (one of the 100 replicates used to generate Table 1 of the main text). All optimizations start from random parameter guesses–initial 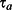 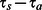 values are chosen uniformly between 0 to 20,000 generations; 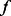 is chosen uniformly on 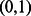; 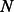 is chosen uniformly between 100 and 100,000. Our numerical routine for finding the optimum of the IBS tract likelihood surface is generally more successful at finding the optimum than the analogous routines that are part of the 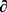
a
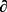
i package.(PDF)

Text S1 **Supporting information.**
(PDF)
