# Supplementary material for: Inferring Demographic History from a Spectrum of Shared Haplotype Lengths
Source: PLoS Genet. 2013 Jun 6;9(6):e1003521. doi: 10.1371/journal.pgen.1003521 (PMC3675002; doi:10.1371/journal.pgen.1003521)
Supplement: Table S1 — Numerical performance of a i optimization vs. IBS tract inference. The left table contains the results of 20 ai Nelder-Mead optimizations attempting to guess demographic parameters from an allele frequency spectrum. There is no population size estimate because ai estimates it analytically before the optimization begins. The right table contains the result of 20 analogous optimizations that use an equivalent amount of IBS tract data (one of the 100 replicates used to generate Table 1 of the main text). All optimizations start from random parameter guesses–initial values are chosen uniformly between 0 to 20,000 generations; is chosen uniformly on ; is chosen uniformly between 100 and 100,000. Our numerical routine for finding the optimum of the IBS tract likelihood surface is generally more successful at finding the optimum than the analogous routines that are part of the ai package. (PDF) [file pgen.1003521.s014.pdf]

Supplementary Table 1: **Numerical stability of ∂a∂i vs. IBS tract inference**

-log likelihood	τ_a	τ_s	f
Simul. params:	0.01	0.1	0.05
8329.9620198	0.03659	0.1055	0.104
26328.1005948	0.06069	0.1120	0.232
26333.3741567	0.06071	0.1120	0.232
27515.9085976	0.05863	0.1106	0.208
40172.3596033	0.08467	0.1106	0.542
45041.8469532	0.08896	0.1291	0.856
45041.8469846	0.08896	0.1291	0.856
66521.0187249	0.09032	0.09040	0.333
66521.0187249	0.09032	0.09040	0.333
66521.0187253	0.09032	0.09040	0.333
66521.18999	0.09035	0.09043	0.668
66521.1899907	0.09035	0.09043	0.668
66521.1899912	0.09035	0.09043	0.668
66521.2117573	0.09032	0.09040	0.327
66841.6311539	0.09037	0.09037	0.849
66841.6916678	0.09037	0.09037	0.849
66847.2382421	0.09037	0.09037	0.896
66849.1579895	0.09037	0.09037	0.911
66851.6329195	0.09037	0.09037	0.0709
66855.8711617	0.09037	0.09037	0.959

-log likelihood	τ_a	$\tau_s - \tau_a$	f	$N \times 10^{-4}$
Simul. params:	0.01	0.09	0.05	1
44.69326588422074	0.01083835	0.08870295	0.05276519	1.00355111
44.693265893441264	0.01083864	0.08870302	0.05276541	1.0035453
44.693265917129885	0.01083854	0.08870251	0.05276585	1.00355611
44.69326593481351	0.01083841	0.08870352	0.05276549	1.00354219
44.69326597041676	0.01083884	0.08870345	0.05276642	1.00353741
44.69326598971337	0.01083823	0.08870245	0.05276505	1.00356078
44.693266033799766	0.01083897	0.0887035	0.05276659	1.00353431
44.693266061466886	0.0108382	0.08870225	0.0527649	1.00356397
44.6932661730406	0.0108392	0.08870364	0.05276679	1.00352958
44.69326620042027	0.01083882	0.08870404	0.05276646	1.00352885
44.69326649533553	0.01083919	0.08870424	0.05276697	1.00352098
44.6932671113402	0.01083957	0.0887046	0.05276771	1.00350983
44.693268280577676	0.01083968	0.08870552	0.05276809	1.00349454
44.693268832064774	0.01083732	0.08870015	0.05276338	1.00360853
44.69326911467046	0.01083995	0.08870584	0.05276843	1.00348585
44.693269115375614	0.01083886	0.08870406	0.05276158	1.00352361
44.69327005738606	0.01083705	0.08869962	0.05276269	1.00361987
44.69327180464335	0.01083675	0.08869895	0.05276187	1.00363333
44.69327369365423	0.01084035	0.08870774	0.05276928	1.00345149
44.693274370560026	0.01083657	0.0886981	0.05276198	1.00364998
