# Supplementary material for: Inferring Demographic History from a Spectrum of Shared Haplotype Lengths
Source: PLoS Genet. 2013 Jun 6;9(6):e1003521. doi: 10.1371/journal.pgen.1003521 (PMC3675002; doi:10.1371/journal.pgen.1003521)
Supplement: Text S1 — Supporting information. (PDF) [file pgen.1003521.s015.pdf]

Supporting Information: Inferring demographic history from a spectrum of shared haplotype lengths

Kelley Harris and Rasmus Nielsen

Contents

1 Derivation of the IBS tract length formula

1.1 One intra-tract recombination

Consider an alignment between sequences from two populations of constant size N that diverged at time τ_s, measured in units of $2N$ generations before the present. In the main text, we computed the frequency $H^{(0)}_{\tau_s}(L)$ of L-base IBS tracts with no historical recombinations. We now proceed to compute the frequency $H^{(1)}_{\tau_s}(L)$ of L-base IBS tracts with a history that contains exactly one recombination event. We must marginalize over two coalescence times t_0, t to the left and right of the historical recombination site, respectively, as well as the time of recombination $t_{(r)}$ and the location $L_{(r)}$ of the recombination site:

$$\begin{aligned}
H^{(1)}_{\tau_s}(L) &= \sum_{L_{(r)}=1}^{L-1} \int_{t_0=\tau_s}^{\infty} \int_{t=\tau_s}^{\infty} \int_{t_{(r)}=0}^{\min(t_0,t)} e^{-t_0} e^{-t_0 L_{(r)}(\rho+\theta)} (1-e^{-t_0\theta}) \cdot \rho e^{-\rho t_{(r)}} && (1)\\
&\quad \cdot e^{-(t-t_{(r)})} \cdot e^{-t(L-L_{(r)})(\rho+\theta)} dt_{(r)} dt dt_0 && (2)\\
&\approx \int_{L_r=1}^{L-1} \int_{t_0=\tau_s}^{\infty} \int_{t=\tau_s}^{\infty} \int_{t_{(r)}=0}^{\min(t_0,t)} e^{-t_0} e^{-t_0 L_r(\rho+\theta)} (1-e^{-t_0\theta}) \cdot \rho e^{-\rho t_{(r)}} && (3)\\
&\quad \cdot e^{-(t-t_{(r)})} \cdot e^{-t(L-L_r)(\rho+\theta)} dt_{(r)} dt dt_0 dL_r && (4)
\end{aligned}$$

In all formulae, the population size N appears as an implicit factor in $\theta = 4N\mu$ and $\rho = 4Nr$.

Marginalizing over the ordering of t_0 and t to evaluate this integral, we find that

$$H^{(1)}_{\tau_s}(L) = e^{-\tau_s L(\rho+\theta)} \log\left(\frac{(1+(L-1)(\rho+\theta)-\rho)(1+(L-1)(\rho+\theta))}{(1+\theta)(1+\rho+\theta)}\right) \quad (5)$$

$$\cdot\frac{1-e^{-\tau_s\rho}}{(\rho+\theta)(2+L(\rho+\theta)-\rho)} \quad (6)$$

$$-e^{-\tau_s(L(\rho+\theta)+\theta)}\log\left(\frac{(1+(L-1)(\rho+\theta))^2}{(1+\theta)(1+\rho+2\theta)}\right) \quad (7)$$

$$\cdot\frac{(1-e^{-\tau_s\rho})}{(\rho+\theta)(2+L(\rho+\theta)-\rho+\theta)} \quad (8)$$

$$+\frac{\rho e^{-\tau_s L(\rho+\theta)}}{(1-\rho)(\rho+\theta)}\left(\log\left(\frac{1+(L-1)(\rho+\theta)}{1+\rho+\theta}\right)\left(\frac{1}{1+L(\rho+\theta)}-\frac{1}{2+L(\rho+\theta)-\rho}\right)\right. \quad (9)$$

$$-\log\left(\frac{1+L(\rho+\theta)-\rho}{1+\rho+2\theta}\right)\left(\frac{e^{-\tau_s\theta}}{1+L(\rho+\theta)+\theta}-\frac{e^{-\tau_s\theta}}{2+L(\rho+\theta)+\theta-\rho}\right) \quad (10)$$

$$+\log\left(\frac{1+(L-1)(\rho+\theta)-\rho}{1+\theta}\right)\left(\frac{1}{1+L(\rho+\theta)}-\frac{e^{-\tau_s\theta}}{1+L(\rho+\theta)+\theta}\right. \quad (11)$$

$$\left.\left.-\frac{1}{2+L(\rho+\theta)-\rho}+\frac{e^{-\tau_s\theta}}{2+L(\rho+\theta)+\theta-\rho}\right)\right) \quad (12)$$

1.2 Two intra-tract recombinations

The accuracy of the approximation $H_{\tau_s}(L) \approx H^{(0)}_{\tau_s}(L) + H^{(1)}_{\tau_s}(L)$ decreases as the mutation rate decreases below the recombination rate, making it desirable to compute the more accurate formula $H_{\tau_s}(L) \approx H^{(0)}_{\tau_s}(L) + H^{(1)}_{\tau_s}(L) + H^{(2)}_{\tau_s}(L)$. Conceptually, computing $H^{(2)}_{\tau_s}(L)$ is no different from computing $H^{(1)}_{\tau_s}(L)$; it simply requires integrating over three coalescence times t_0, t_1, t_2, two recombination times $t_{(r,1)} < \min(t_0, t_1), t_{(r,2)} < \min(t_1, t_2)$, and the locations $L_1 < L_2$ of two distinct recombination sites:

$$H^{(2)}_{\tau_s}(L) = \int_{L_1=1}^{L-2}\int_{L_2=1}^{L-L_1-1}\int_{t_0=\tau_s}^{\infty}\int_{t_1=\tau_s}^{\infty}\int_{t_2=\tau_s}^{\infty}\int_{t_{(r,1)}=0}^{\min(t_0,t_1)}\int_{t_{(r,2)}=0}^{\min(t_1,t_2)} e^{-t_0}e^{-t_0L_1(\rho+\theta)} \quad (13)$$

$$\cdot(1-e^{-t_0\theta})\cdot\rho e^{-\rho t_{(r,1)}}\cdot e^{-(t_1-t_{(r,1)})}\cdot e^{-t_1L_1(\rho+\theta)}\cdot\rho e^{-\rho t_{(r,2)}}\cdot e^{-(t_2-t_{(r,2)})} \quad (14)$$

$$\cdot e^{-t_2(L-L_1-L_2)(\rho+\theta)}\; dt_{(r,2)}dt_{(r,1)}dt_2dt_1dt_0dL_2dL_1 \quad (15)$$

The result is that

$$H^{(2)}_{\tau_s}(L) = \left(\frac{\rho}{\rho+\theta}\right)^2\left(e^{-\tau_s L(\rho+\theta)}(2p_1-p_2-p_3) - e^{-\tau_s(L(\rho+\theta)+\theta)}(2p_4-p_5-p_6)\right),$$

where

$$
\begin{aligned}
p_1 \; = \; & \frac{1}{(1-\rho)(1+L(\rho+\theta))(2-\rho+L(\rho+\theta))}\left(\log\left(\frac{\rho+(L-2)(\rho+\theta)}{2\rho+\theta}\right)\log(1+(L-1)(\rho+\theta))\right. \\
& -\log(1+\theta)\log\left(\frac{1-\rho+(L-1)(\rho+\theta)}{1+2\rho+\theta}\right) \\
& \left.-\mathrm{Li}\left(\frac{\rho+(L-2)(\rho+\theta)}{1+(L-1)(\rho+\theta)}\right)+\mathrm{Li}\left(\frac{2\rho+\theta}{1+(L-1)(\rho+\theta)}\right)-\log(1+\theta)\log\left(\frac{\rho+(L-2)(\rho+\theta)}{2\rho+\theta}\right)\right) \\
& +\frac{1}{(1-\rho)(3-2\rho+L(\rho+\theta))(2-\rho+L(\rho+\theta))}(\\
& -\log(2+\theta+(L-2)(\rho+\theta))\log\left(\frac{(1+(L-2)(\rho+\theta)}{1+\rho+\theta}\right) \\
& +\mathrm{Li}\left(\frac{1+(L-2)(\rho+\theta)}{2+\theta+(L-2)(\rho+\theta)}\right)-\mathrm{Li}\left(\frac{1+\rho+\theta}{2+\theta+(L-2)(\rho+\theta)}\right) \\
& -\log(1+\theta)\log\frac{1+\rho+2\theta}{\rho+\theta}+\log(1+\theta+(L-3)(\rho+\theta))\log\left(\frac{1+\theta+(L-2)(\rho+\theta)}{\rho+\theta}\right) \\
& -\mathrm{Li}\left(-\frac{1+\theta+(L-3)(\rho+\theta)}{1+\theta}\right)-\log(1+\theta+(L-3)(\rho+\theta))\log\left(\frac{2-2\rho+(L-1)(\rho+\theta)}{1+\theta}\right) \\
& +\mathrm{Li}(-1)+\log(1+t)\log(2)+\log(1+\theta)\log\left(\frac{2-2\rho+(L-1)(\rho+\theta)}{2+2\theta}\right) \\
& \left.-\mathrm{Li}\left(-\frac{1+\theta}{\rho+\theta}\right)+\mathrm{Li}\left(-\frac{1+\theta+(L-3)(\rho+\theta)}{\rho+\theta}\right)\right)
\end{aligned}
$$

$$
\begin{aligned}
p_2 \; = \; & \frac{1}{(1-\rho)(1+L(\rho+\theta))(2-\rho+L(\rho+\theta))}\left(\log(1+\theta)\log\left(\frac{1+(L-2)(\rho+\theta)}{1+\rho+\theta}\right)\right. \\
& -\log(2+\theta+(L-2)(\rho+\theta))\log\left(\frac{1+(L-2)(\rho+\theta)}{1+\rho+\theta}\right)+\mathrm{Li}\left(\frac{1+(L-2)(\rho+\theta)}{2+\theta+(L-2)(\rho+\theta)}\right) \\
& -\mathrm{Li}\left(\frac{1+\rho+\theta}{2+\theta+(L-2)(\rho+\theta)}\right)+\log(1+(L-1)(\rho+\theta))\log\left(\frac{r+(L-2)(\rho+\theta)}{2\rho+\theta}\right) \\
& \left.+\mathrm{Li}\left(\frac{2\rho+\theta}{1+(L-1)(\rho+\theta)}\right)-\mathrm{Li}\left(\frac{\rho+(L-2)(\rho+\theta)}{1+(L-1)(\rho+\theta)}\right)-\log(1+\theta)\log\left(\frac{\rho+(L-2)(\rho+\theta)}{2\rho+\theta}\right)\right)
\end{aligned}
$$

$$
\begin{aligned}
p_3 \;=\;& \frac{1}{(1-\rho)(1+L(\rho+\theta))(2-\rho+L(\rho+\theta))}\left(\log(1+(L-1)(\rho+\theta))\log\left(\frac{\rho+(L-2)(\rho+\theta)}{2\rho+\theta}\right)\right.\\
&-\log(1+\theta)\log\left(\frac{\rho+(L-2)(\rho+\theta)}{2\rho+\theta}\right)+\mathrm{Li}\left(-\frac{\rho+(L-2)(\rho+\theta)}{1+\theta}\right)-\mathrm{Li}\left(-\frac{2\rho+\theta}{1+\theta}\right)\\
&-\log(1+(L-1)(\rho+\theta))\log\left(\frac{1+(L-2)(\rho+\theta)}{1+\rho+\theta}\right)+\log(\rho+\theta)\log\left(\frac{1+(L-2)(\rho+\theta)}{1+\rho+\theta}\right)\\
&\left.-\mathrm{Li}\left(-\frac{1+(L-2)(\rho+\theta)}{\rho+\theta}\right)+\mathrm{Li}\left(-\frac{1+r+t}{r+t}\right)\right)
\end{aligned}
$$

$$
\begin{aligned}
p_4 \;=\;& \frac{1}{(1-\rho)(1+\theta+L(\rho+\theta))(2-\rho+\theta+L(\rho+\theta))}\left(\log\left(\frac{L-1}{2}\right)\log(1+\theta+(L-1)(\rho+\theta))\right.\\
&-\log(1+\theta)\log\left(\frac{1+\rho+2\theta}{\rho+\theta}\right)+\log(1+\theta+(L-3)(\rho+\theta))\log\left(\frac{1+\theta+(L-2)(\rho+\theta)}{\rho+\theta}\right)\\
&-\mathrm{Li}\left(-\frac{1+\theta}{\rho+\theta}\right)+\mathrm{Li}\left(-\frac{1+\theta+(L-3)(\rho+\theta)}{\rho+\theta}\right)-\log(1+\theta)\log\left(\frac{1-\rho+(L-1)(\rho+\theta)}{1+2\rho+\theta}\right)\\
&\left.-\mathrm{Li}\left(\frac{(L-1)(\rho+\theta)}{1+\theta+(L-1)(\rho+\theta)}\right)+\mathrm{Li}\left(\frac{2(\rho+\theta)}{1+\theta+(L-1)(\rho+\theta)}\right)-\log(1+\theta)\log\left(\frac{L-1}{2}\right)\right)\\
&+\frac{1}{(1-\rho)(3-2\rho+\theta+L(\rho+\theta))(2-\rho+\theta+L(\rho+\theta))}\left(\mathrm{Li}\left(\frac{1+\theta+(L-2)(\rho+\theta)}{2+2\theta+(L-2)(\rho+\theta)}\right)\right.\\
&-\log(2+2\theta+(L-2)(\rho+\theta))\log\left(\frac{1+\theta+(L-2)(\rho+\theta)}{1+2\theta+\rho}\right)\\
&-\mathrm{Li}\left(\frac{1+2\theta+\rho}{2+2\theta+(L-2)(\rho+\theta)}\right)+\log(1+\theta)\log\left(\frac{1+\theta+(L-2)(\rho+\theta)}{1+2\theta+\rho}\right)\\
&-\mathrm{Li}\left(-\frac{1+\theta+(L-3)(\rho+\theta)}{1+\theta}\right)-\log(1+\theta+(L-3)(\rho+\theta))\log\left(\frac{2-2\rho+(L-1)(\rho+\theta)}{1+\theta}\right)\\
&\left.+\mathrm{Li}(-1)+\log(1+\theta)\log(2)+\log(1+\theta)\log\left(\frac{2-2\rho+(L-1)(\rho+\theta)}{2+2\theta}\right)\right)
\end{aligned}
$$

$$
\begin{aligned}
p_5 \;=\;& \frac{1}{(1-\rho)(2-\rho+\theta+L(\rho+\theta))(1+\theta+L(\rho+\theta))}\left(\mathrm{Li}\left(\frac{1+\theta+(L-2)(\rho+\theta)}{2+2\theta+(L-2)(\rho+\theta)}\right)\right.\\
&-\log(2+2\theta+(L-2)(\rho+\theta))\log\left(\frac{1+\theta+(L-2)(\rho+\theta)}{1+2\theta+\rho}\right)-\mathrm{Li}\left(\frac{1+2\theta+\rho}{2+2\theta+(L-2)(\rho+\theta)}\right)\\
&+\log(1+\theta)\log\left(\frac{1+\theta+(L-2)(\rho+\theta)}{1+2\theta+\rho}\right)+\log(1+\theta+(L-1)(\rho+\theta))\log\left(\frac{L-1}{2}\right)\\
&\left.+\mathrm{Li}\left(\frac{2(\rho+\theta)}{1+\theta+(L-1)(\rho+\theta)}\right)-\mathrm{Li}\left(\frac{(L-1)(\rho+\theta)}{1+\theta+(L-1)(\rho+\theta)}\right)-\log(1+\theta)\log\left(\frac{L-1}{2}\right)\right)
\end{aligned}
$$

$$p_6 = \frac{1}{(1-\rho)(2-\rho+\theta+L(\rho+\theta))(1+\theta+L(\rho+\theta))}\left(\log(1+\theta+(L-1)(\rho+\theta))\log\left(\frac{L-1}{2}\right)\right.$$
$$-\log(1+\theta)\log\left(\frac{L-1}{2}\right)+\mathrm{Li}\left(-\frac{(L-1)(\rho+\theta)}{1+\theta}\right)-\mathrm{Li}\left(-\frac{2(\rho+\theta)}{1+\theta}\right)$$
$$-\log(1+\theta+(L-1)(\rho+\theta))\log\left(\frac{1+\theta+(L-2)(\rho+\theta)}{1+2\theta+\rho}\right)+\mathrm{Li}\left(-\frac{1+\rho+2\theta}{\rho+\theta}\right)$$
$$\left.+\log(\rho+\theta)\log\left(\frac{1+\theta+(L-2)(\rho+\theta)}{1+2\theta+\rho}\right)-\mathrm{Li}\left(-\frac{1+\theta+(L-2)(\rho+\theta)}{\rho+\theta}\right)\right)$$

To approximate the polylog function with elementary functions, we make use of the fact that

$$\lim_{z\to\infty}\frac{\mathrm{Li}(-z)}{-\pi^2/6-\log(z)^2/2}=1 \tag{16}$$

and

$$\mathrm{Li}(-1+\epsilon)=-\frac{\pi^2}{12}+\epsilon\log(2)+O(\epsilon^2). \tag{17}$$

Specifically, we use (**??**) to approximate $\mathrm{Li}(z)$ for $z<-4$ and (**??**) to approximate $\mathrm{Li}(z)$ for $z>-2$. For the intermediate regime, we use the unique cubic polynomial that joins up with the left and right halves to form a function with continuous first derivative.

1.3 Mixed admixture status

Consider a history described by four parameters: a population size N, a split time τ_s, a later admixture time τ_a and an admixture fraction f that denotes the percentage of individuals in the recipient population that recently migrated over from the donor population. Given this history, we will describe how to calculate the frequency $H^{(1)}_{\tau_a,\tau_s,f}(L)$ of IBS tracts with one internal recombination.

As with previous calculations, we must marginalize over two coalescence times t_0 and t, as well as a time of recombination $t_{(r)}<\min(t_0,t)$. In addition, we must consider the "admixture status" of each tract half: whether one of the sequences was involved in the historical migration or whether they were constrained to coalesce before the split time. We will say that a locus is admixture-positive if one of the sequences was involved in the migration and admixture-negative otherwise (see Figure S13). This allows us to introduce the conditional coalescence density function $\zeta(t|(t_{(r)},a))$, which denotes the coalescence time density function given that at time $t_{(r)}$, the base pair was uncoalesced with admixture status a. When $t_{(r)}<\tau_a$, the admixture status at the time of recombination is undetermined, which will be denoted '0'. Conversely, the admixture status cannot be undetermined at more ancient times of recombination $t_{(r)}>\tau_a$. For the one-pulse, constant size history considered here, $\zeta(t|(t_{(r)},a))$ is the following:

$$\zeta(t|(t_{(r)},0)) = \zeta(t) = fe^{-(t-\tau_a)}\mathbf{1}(t \geq \tau_a, t_{(r)} < \tau_a) + (1-f)e^{-(t-\tau_s)}\mathbf{1}(t \geq \tau_s, t_{(r)} < \tau_a) \quad (18)$$

$$\zeta(t|(t_{(r)},+)) = e^{-(t-t_{(r)})}\mathbf{1}(\tau_a \leq t_{(r)} < t) \quad (19)$$

$$\zeta(t|(t_{(r)},-) = e^{-(t-\max(t_{(r)},\tau_s))}\mathbf{1}(\tau_a \leq t_{(r)} < t) \quad (20)$$

If we let a denote the admixture status at the time of recombination, then

$$H^{(1)}_{\tau_a,\tau_s,f}(L) = \sum_{L_r=1}^{L-1}\sum_{a\in\{+,-,0\}}\int_{t_0=0}^{\infty}\int_{t=0}^{\infty}\int_{t_{(r)}=0}^{\min(t_0,t)}\zeta(t_0)e^{-t_0L_r(\rho+\theta)}(1-e^{-t_0\theta})\cdot\rho e^{-\rho t_{(r)}} \quad (21)$$

$$\cdot\mathbb{P}(a|t_0,t_{(r)})\cdot\zeta(t|(t_{(r)},a))e^{-t(L-L_r)(\rho+\theta)+t\rho}dt_{(r)}dtdt_0 \quad (22)$$

$$\approx \sum_{a\in\{+,-,0\}}\int_{L_r=1}^{L-1}\int_{t_0=0}^{\infty}\int_{t=0}^{\infty}\int_{t_{(r)}=0}^{\min(t_0,t)}\zeta(t_0)e^{-t_0L_r(\rho+\theta)}(1-e^{-t_0\theta})\cdot\rho e^{-\rho t_{(r)}} \quad (23)$$

$$\cdot\mathbb{P}(a|t_0,t_{(r)})\cdot\zeta(t|(t_{(r)},a))e^{-t(L-L_r)(\rho+\theta)+t\rho}dL_rdt_{(r)}dtdt_0. \quad (24)$$

Therefore, our goal is to evaluate the expression

$$H^{(1)}_{\tau_a,\tau_s,f}(L) = \sum_{a\in\{+,-,0\}}\int_{L_r=1}^{L-1}\int_{t_0=0}^{\infty}\int_{t=0}^{\infty}\int_{t_{(r)}=0}^{\min(t_0,t)}\zeta(t_0)e^{-t_0L_r(\rho+\theta)}(1-e^{-t_0\theta})\cdot\rho e^{-\rho t_{(r)}} \quad (25)$$

$$\cdot\mathbb{P}(a|t_0,t_{(r)})\cdot\zeta(t|(t_{(r)},a))e^{-t(L-L_r)(\rho+\theta)+t\rho}dL_rdt_{(r)}dtdt_0 \quad (26)$$

in closed form. Marginalizing over the admixture status at the time of recombination yields that

$$H^{(1)}_{\tau_a,\tau_s,f}(L) = \int_{L_r=1}^{L-1}\int_{t_0=0}^{\infty}\int_{t=0}^{\infty}\int_{t_{(r)}=0}^{\tau_a}(fe^{-(t_0-\tau_a)}\mathbf{1}(t_0\geq\tau_a)+(1-f)e^{-(t_0-\tau_s)}\mathbf{1}(t_0\geq\tau_s)) \quad (27)$$

$$\cdot e^{-t_0L_r(\rho+\theta)}(1-e^{-t_0\theta})\cdot\rho e^{-\rho t_{(r)}}\cdot(fe^{-(t-\tau_a)}\mathbf{1}(t\geq\tau_a)+(1-f)e^{-(t-\tau_s)}\mathbf{1}(t\geq\tau_s)) \quad (28)$$

$$\cdot e^{-t(L-L_r)(\rho+\theta)+t\rho}dL_rdt_{(r)}dtdt_0 \quad (29)$$

$$+\int_{L_r=1}^{L-1}\int_{t_0=\tau_a}^{\infty}\int_{t=\tau_a}^{\infty}\int_{t_{(r)}=\tau_a}^{\min(t_0,t)}fe^{-(t_0-\tau_a)}e^{-t_0L_r(\rho+\theta)}(1-e^{-t_0\theta})\cdot\rho e^{-\rho t_{(r)}} \quad (30)$$

$$\cdot e^{-(t-t_{(r)})}e^{-t(L-L_r)(\rho+\theta)+t\rho}dL_rdt_{(r)}dtdt_0 \quad (31)$$

$$+\int_{L_r=1}^{L-1}\int_{t_0=\tau_s}^{\infty}\int_{t=\tau_s}^{\infty}\int_{t_{(r)}=\tau_a}^{\min(t_0,t)}(1-f)e^{-(t_0-\tau_a)}e^{-t_0L_r(\rho+\theta)}(1-e^{-t_0\theta})\cdot\rho e^{-\rho t_{(r)}} \quad (32)$$

$$\cdot e^{-(t-\max(\tau_s,t_{(r)}))}e^{-t(L-L_r)(\rho+\theta)+t\rho}dL_rdt_{(r)}dtdt_0 \quad (33)$$

$$(34)$$

The easiest integrals to compute are the ones where the whole segment has the same admixture status. These scenarios split into two classes: one where the recombination is more recent than τ_a (class R) and one where the one where the recombination is more ancient than τ_a (class A). When admixture status is negative it also matters whether recombination happened before or after τ_s, which divides class A into two subclasses A_i (intermediate recombination times, $\tau_a \leq t_{(r)} < \tau_s$) and A_a (ancient recombination times, $t_{(r)} \geq \tau_s$). The scenarios with mixed admixture status involve exponential integrals, which cannot be computed in closed form and must be approximated further. In total, there are seven integrals we must do:

$$\begin{aligned} H^{(1)}_{\tau_a,\tau_s,f}(L) &= H^{(1)}_{\tau_a,\tau_s,f}(L,+,R,+) + H^{(1)}_{\tau_a,\tau_s,f}(L,+,A,+) + H^{(1)}_{\tau_a,\tau_s,f}(L,-,R,-) \\ &+ H^{(1)}_{\tau_a,\tau_s,f}(L,-,A_i,-) + H^{(1)}_{\tau_a,\tau_s,f}(L,-,A_a,-) + H^{(1)}_{\tau_a,\tau_s,f}(L,+,R,-) \\ &+ H^{(1)}_{\tau_a,\tau_s,f}(L,-,R,+), \end{aligned} \tag{35}$$

where

$$H^{(1)}_{\tau_a,\tau_s,f}(L,+,R,+) := \int_{L_r=1}^{L-1}\int_{t_0=\tau_a}^{\infty}\int_{t=\tau_a}^{\infty}\int_{t_{(r)}=0}^{\tau_a} fe^{-(t_0-\tau_a)}\cdot e^{-t_0L_r(\rho+\theta)}(1-e^{-t_0\theta})\cdot\rho e^{-\rho t_{(r)}} \tag{36}$$

$$\cdot fe^{-(t-\tau_a)}\cdot e^{-t(L-L_r)(\rho+\theta)+t\rho}dL_r dt_{(r)}dtdt_0 \tag{37}$$

$$H^{(1)}_{\tau_a,\tau_s,f}(L,+,A,+) := \int_{L_r=1}^{L-1}\int_{t_0=\tau_a}^{\infty}\int_{t=\tau_a}^{\infty}\int_{t_{(r)}=\tau_a}^{\min(t_0,t)} fe^{-(t_0-\tau_a)}\cdot e^{-t_0L_r(\rho+\theta)}(1-e^{-t_0\theta})\cdot\rho e^{-\rho t_{(r)}} \tag{38}$$

$$\cdot e^{-(t-t_{(r)})}\cdot e^{-t(L-L_r)(\rho+\theta)+t\rho}dL_r dt_{(r)}dtdt_0 \tag{39}$$

$$H^{(1)}_{\tau_a,\tau_s,f}(L,-,R,-) := \int_{L_r=1}^{L-1}\int_{t_0=\tau_s}^{\infty}\int_{t=\tau_s}^{\infty}\int_{t_{(r)}=0}^{\tau_a}(1-f)e^{-(t_0-\tau_s)}\cdot e^{-t_0L_r(\rho+\theta)}(1-e^{-t_0\theta})\cdot\rho e^{-\rho t_{(r)}} \tag{40}$$

$$\cdot(1-f)e^{-(t-\tau_s)}\cdot e^{-t(L-L_r)(\rho+\theta)+t\rho}dL_r dt_{(r)}dtdt_0 \tag{41}$$

$$H^{(1)}_{\tau_a,\tau_s,f}(L,-,A_i,-) := \int_{L_r=1}^{L-1}\int_{t_0=\tau_s}^{\infty}\int_{t=\tau_s}^{\infty}\int_{t_{(r)}=\tau_a}^{\tau_s}(1-f)e^{-(t_0-\tau_s)}\cdot e^{-t_0L_r(\rho+\theta)}(1-e^{-t_0\theta})\cdot\rho e^{-\rho t_{(r)}} \tag{42}$$

$$\cdot e^{-(t-\tau_s)}\cdot e^{-t(L-L_r)(\rho+\theta)+t\rho}dL_r dt_{(r)}dtdt_0 \tag{43}$$

$$H^{(1)}_{\tau_a,\tau_s,f}(L,-,A_a,-) := \int_{L_r=1}^{L-1}\int_{t_0=\tau_s}^{\infty}\int_{t=\tau_s}^{\infty}\int_{t_{(r)}=\tau_s}^{\min(t_0,t)}(1-f)e^{-(t_0-\tau_s)}\cdot e^{-t_0L_r(\rho+\theta)}(1-e^{-t_0\theta})\cdot\rho e^{-\rho t_{(r)}} \tag{44}$$

$$\cdot e^{-(t-\tau_s)}\cdot e^{-t(L-L_r)(\rho+\theta)+t\rho}dL_r dt_{(r)}dtdt_0 \tag{45}$$

$$H^{(1)}_{\tau_a,\tau_s,f}(L,+,R,-) := \int_{L_r=1}^{L-1}\int_{t_0=\tau_a}^{\infty}\int_{t=\tau_s}^{\infty}\int_{t_{(r)}=0}^{\tau_a} fe^{-(t_0-\tau_a)}\cdot e^{-t_0L_r(\rho+\theta)}(1-e^{-t_0\theta})\cdot\rho e^{-\rho t_{(r)}} \tag{46}$$

$$\cdot(1-f)e^{-(t-\tau_s)}\cdot e^{-t(L-L_r)(\rho+\theta)+t\rho}dL_r dt_{(r)}dtdt_0 \tag{47}$$

$$H^{(1)}_{\tau_a,\tau_s,f}(L,-,R,+) := \int_{L_r=1}^{L-1}\int_{t_0=\tau_s}^{\infty}\int_{t=\tau_a}^{\infty}\int_{t_{(r)}=0}^{\tau_a}(1-f)e^{-(t_0-\tau_s)}\cdot e^{-t_0L_r(\rho+\theta)}(1-e^{-t_0\theta})\cdot\rho e^{-\rho t_{(r)}} \tag{48}$$

$$\cdot fe^{-(t-\tau_a)}\cdot e^{-t(L-L_r)(\rho+\theta)+t\rho}dL_r dt_{(r)}dtdt_0 \tag{49}$$

The first five of these integrals can be evaluated in closed form with no further approxima-

tion. After some algebra, we find that

$$H^{(1)}_{\tau_a,\tau_s,f}(L,+,R,+) = f^2 e^{-\tau_a L(\rho+\theta)} \log\left(\frac{(1+(L-1)(\rho+\theta)-\rho)(1+(L-1)(\rho+\theta)}{(1+\theta)(1+\rho+\theta)}\right) \tag{50}$$

$$\cdot\frac{1-e^{-\tau_a\rho}}{(\rho+\theta)(2+L(\rho+\theta)-\rho)} \tag{51}$$

$$-f^2 e^{-\tau_a(L(\rho+\theta)+\theta)} \log\left(\frac{(1+(L-1)(\rho+\theta))^2}{(1+\theta)(1+\rho+2\theta)}\right) \tag{52}$$

$$\cdot\frac{(1-e^{-\tau_a\rho})}{(\rho+\theta)(2+L(\rho+\theta)-\rho+\theta)} \tag{53}$$

$$H^{(1)}_{\tau_a,\tau_s,f}(L,+,A,+) = \frac{f\rho e^{-\tau_a L(\rho+\theta)}}{(1-\rho)(\rho+\theta)}\left(\log\left(\frac{1+(L-1)(\rho+\theta)}{1+\rho+\theta}\right)\left(\frac{1}{1+L(\rho+\theta)}-\frac{1}{2+L(\rho+\theta)-\rho}\right)\right. \tag{54}$$

$$-\log\left(\frac{1+L(\rho+\theta)-\rho}{1+\rho+2\theta}\right)\left(\frac{e^{-\tau_a\theta}}{1+L(\rho+\theta)+\theta}-\frac{e^{-\tau_a\theta}}{2+L(\rho+\theta)+\theta-\rho}\right) \tag{55}$$

$$+\log\left(\frac{1+(L-1)(\rho+\theta)-\rho}{1+\theta}\right)\left(\frac{1}{1+L(\rho+\theta)}-\frac{e^{-\tau_a\theta}}{1+L(\rho+\theta)+\theta}\right. \tag{56}$$

$$\left.\left.-\frac{1}{2+L(\rho+\theta)-\rho}+\frac{e^{-\tau_a\theta}}{2+L(\rho+\theta)+\theta-\rho}\right)\right) \tag{57}$$

$$H^{(1)}_{\tau_a,\tau_s,f}(L,-,R,-) = (1-f)^2 e^{-\tau_s L(\rho+\theta)} \log\left(\frac{(1+(L-1)(\rho+\theta)-\rho)(1+(L-1)(\rho+\theta)}{(1+\theta)(1+\rho+\theta)}\right) \tag{58}$$

$$\cdot\frac{1-e^{-\tau_a\rho}}{(\rho+\theta)(2+L(\rho+\theta)-\rho)} \tag{59}$$

$$-(1-f)^2 e^{-\tau_s(L(\rho+\theta)+\theta)} \log\left(\frac{((1+(L-1)(\rho+\theta)-\rho)(1+(L-1)(\rho+\theta))}{(1+\theta)(1+\rho+2\theta)}\right) \tag{60}$$

$$\cdot\frac{(1-e^{-\tau_a\rho})}{(\rho+\theta)(2+L(\rho+\theta)-\rho+\theta)} \tag{61}$$

$$H^{(1)}_{\tau_a,\tau_s,f}(L,-,A_i,-) = (1-f)e^{-\tau_s L(\rho+\theta)} \log\left(\frac{(1+(L-1)(\rho+\theta)-\rho)(1+(L-1)(\rho+\theta)}{(1+\theta)(1+\rho+\theta)}\right) \tag{62}$$

$$\cdot\frac{e^{-\tau_a\rho}-e^{-\tau_s\rho}}{(\rho+\theta)(2+L(\rho+\theta)-\rho)} \tag{63}$$

$$-(1-f)e^{-\tau_a(L(\rho+\theta)+\theta)} \log\left(\frac{((1+(L-1)(\rho+\theta)-\rho)(1+(L-1)(\rho+\theta))}{(1+\theta)(1+\rho+2\theta)}\right) \tag{64}$$

$$\cdot\frac{e^{-\tau_a\rho}-e^{-\tau_s\rho}}{(\rho+\theta)(2+L(\rho+\theta)-\rho+\theta)} \tag{65}$$

$$\tag{66}$$

$$H^{(1)}_{\tau_a,\tau_s,f}(L,-,A_a,-) = \frac{(1-f)\rho e^{-\tau_s L(\rho+\theta)}}{(1-\rho)(\rho+\theta)}\left(\log\left(\frac{1+(L-1)(\rho+\theta)}{1+\rho+\theta}\right)\left(\frac{1}{1+L(\rho+\theta)}-\frac{1}{2+L(\rho+\theta)-\rho}\right)\right. \tag{67}$$

$$-\log\left(\frac{1+L(\rho+\theta)-\rho}{1+\rho+2\theta}\right)\left(\frac{e^{-\tau_s\theta}}{1+L(\rho+\theta)+\theta}-\frac{e^{-\tau_s\theta}}{2+L(\rho+\theta)+\theta-\rho}\right) \tag{68}$$

$$+\log\left(\frac{1+(L-1)(\rho+\theta)-\rho}{1+\theta}\right)\left(\frac{1}{1+L(\rho+\theta)}-\frac{e^{-\tau_s\theta}}{1+L(\rho+\theta)+\theta}\right. \tag{69}$$

$$\left.\left.-\frac{1}{2+L(\rho+\theta)-\rho}+\frac{e^{-\tau_s\theta}}{2+L(\rho+\theta)+\theta-\rho}\right)\right) \tag{70}$$

$$\tag{71}$$

The last terms $H^{(1)}_{\tau_a,\tau_s,f}(L,+,R,-)$ and $H^{(1)}_{\tau_a,\tau_s,f}(L,-,R,+)$, as mentioned before, involve exponential integrals that cannot be reduced to elementary functions. We will approximate them using a standard tight bracketing of the exponential integral. Since

$$\frac{1}{2}e^{-x}\log\left(1+\frac{2}{x}\right) < \int_x^\infty \frac{e^{-\tau}}{\tau}d\tau < e^{-x}\log\left(1+\frac{1}{x}\right), \tag{72}$$

(see [?]), we will let

$$\in \tau_a^b\frac{e^{-\tau}}{\tau}d\tau \approx e^{-a}\log\left(1+\frac{1}{a}\right)-\frac{1}{2}e^{-b}\log\left(1+\frac{2}{b}\right) \tag{73}$$

and use this to derive the approximation

$$\begin{aligned}
H^{(1)}_{\tau_a,\tau_s,f}(L,-,R,+) &= \frac{f(1-f)(1-e^{-\tau_a\rho})}{\rho+\theta}\Bigg(\frac{1}{2+L(\rho+\theta)-\rho}\Big(\\
& e^{\tau_s-\tau_a(1+L(\rho+\theta)-\rho)}\int_{u=(\tau_s-\tau_a)(1+\rho+\theta)}^{(\tau_s-\tau_a)(1+(L-1)(\rho+\theta))}\frac{e^{-u}}{u}du \\
& + e^{\tau_a-\tau_s(1+L(\rho+\theta)-\rho)}\int_{u=(\tau_s-\tau_a)(1+\theta)}^{(\tau_s-\tau_a)(1+(L-1)(\rho+\theta)-\rho)}\frac{e^{u}}{u}du\Big) \\
& -\frac{1}{2+L(\rho+\theta)+\theta-\rho}\Bigg(e^{\tau_s-\tau_a(1+L(\rho+\theta)+\theta-\rho)}\int_{u=(\tau_s-\tau_a)(1+2\theta+\rho)}^{(\tau_s-\tau_a)(1+L(\rho+\theta)-\rho)}\frac{e^{-u}}{u}du \\
& +e^{\tau_a-\tau_s(1+L(\rho+\theta)+\theta-\rho)}\int_{u=(\tau_s-\tau_a)(1+\theta)}^{(\tau_s-\tau_a)(1+(L-1)(\rho+\theta)-\rho)}\frac{e^{u}}{u}du\Bigg)\Bigg)\Bigg) \\
&\approx \frac{f(1-f)(1-e^{-\tau_a\rho})}{\rho+\theta}\Bigg(\frac{1}{2+L(\rho+\theta)-\rho}\Big(\\
& e^{\tau_s-\tau_a(1+L(\rho+\theta)-\rho)}\Bigg(e^{-(\tau_s-\tau_a)(1+\rho+\theta)}\log\left(1+\frac{1}{(\tau_s-\tau_a)(1+\rho+\theta)}\right) \\
& -\frac{1}{2}e^{-(\tau_s-\tau_a)(1+(L-1)(\rho+\theta))}\log\left(1+\frac{2}{(\tau_s-\tau_a)(1+(L-1)(\rho+\theta))}\right)\Bigg) \\
& +e^{\tau_a-\tau_s(1+L(\rho+\theta)-\rho)}\Bigg(e^{(\tau_s-\tau_a)(1+(L-1)(\rho+\theta)-\rho)}\log\left(1+\frac{1}{(\tau_s-\tau_a)(1+(L-1)(\rho+\theta)-\rho}\right) \\
& -\frac{1}{2}e^{(\tau_s-\tau_a)(1+\theta)}\log\left(1+\frac{2}{(\tau_s-\tau_a)(1+\theta)}\right)\Bigg) \\
& -\frac{1}{2+L(\rho+\theta)+\theta-\rho}\Big(e^{\tau_s-\tau_a(1+L(\rho+\theta)+\theta-\rho)} \\
& \Bigg(e^{-(\tau_s-\tau_a)(1+2\theta+\rho)}\log\left(1+\frac{1}{(\tau_s-\tau_a)(1+2\theta+\rho)}\right) \\
& -\frac{1}{2}e^{-(\tau_s-\tau_a)(1+L(\rho+\theta)-\rho)}\log\left(1+\frac{2}{1+L(\rho+\theta)-\rho)}\right)\Bigg) \\
& +e^{\tau_a-\tau_s(1+L(\rho+\theta)-\rho)}\Big(e^{(\tau_s-\tau_a)(1+(L-1)(\rho+\theta)-\rho)} \\
& \cdot\log\left(1+\frac{1}{(\tau_s-\tau_a)(1+(L-1)(\rho+\theta)-\rho)}\right) \\
& -\frac{1}{2}e^{(\tau_s-\tau_a)(1+\theta)}\log\left(1+\frac{2}{1+\theta}\right)\Big)\Big)\Big)\Bigg),
\end{aligned}$$

which simplifies to

$$
\begin{aligned}
H^{(1)}_{\tau_a,\tau_s,f}(L,-,R,+) \quad = \quad & \frac{f(1-f)(1-e^{-\tau_a\rho})}{\rho+\theta}\left(\frac{e^{-\tau_a(L(\rho+\theta)-\rho)}}{2+L(\rho+\theta)-\rho}\right(\\
& e^{-(\tau_s-\tau_a)(\rho+\theta)}\log\left(1+\frac{1}{(\tau_s-\tau_a)(1+\rho+\theta)}\right)\\
& -\frac{1}{2}e^{-(\tau_s-\tau_a)(L-1)(\rho+\theta)}\log\left(1+\frac{2}{(\tau_s-\tau_a)(1+(L-1)(\rho+\theta))}\right)\\
& +e^{-(\tau_s-\tau_a)(\rho+\theta)}\log\left(1+\frac{1}{(\tau_s-\tau_a)(1+(L-1)(\rho+\theta)-\rho)}\right)\\
& -\frac{1}{2}e^{-(\tau_s-\tau_a)(L-1)(\rho+\theta)}\log\left(1+\frac{2}{(\tau_s-\tau_a)(1+\theta)}\right)\Bigg)\\
& -\frac{e^{-\tau_a(L(\rho+\theta)+\theta-\rho)}}{2+L(\rho+\theta)+\theta-\rho}\left(e^{-(\tau_s-\tau_a)(2\theta+\rho)}\log\left(1+\frac{1}{(\tau_s-\tau_a)(1+2\theta+\rho)}\right)\right.\\
& -\frac{1}{2}e^{-(\tau_s-\tau_a)(L(\rho+\theta)-\rho)}\log\left(1+\frac{2}{(\tau_s-\tau_a)(1+L(\rho+\theta)-\rho)}\right)\\
& +e^{-(\tau_s-\tau_a)(2\theta+\rho)}\log\left(1+\frac{1}{(\tau_s-\tau_a)(1+(L-1)(\rho+\theta)-\rho)}\right)\\
& \left.-\frac{1}{2}e^{-(\tau_s-\tau_a)(L(\rho+\theta)-\rho)}\log\left(1+\frac{2}{(\tau_s-\tau_a)(1+\theta)}\right)\right)\Bigg).
\end{aligned}
$$

We can approximate $H^{(1)}_{\tau_a,\tau_s,f}(L,+,R,-)$ in the exact same way:

$$
\begin{aligned}
H^{(1)}_{\tau_a,\tau_s,f}(L,+,R,-) &= \frac{f(1-f)(1-e^{-\tau_a\rho})}{\rho+\theta}\Bigg(\frac{1}{2+L(\rho+\theta)-\rho}\Bigg(\\
&e^{\tau_s-\tau_a(1+L(\rho+\theta)-\rho)}\int_{u=(\tau_s-\tau_a)(1+\theta)}^{(\tau_s-\tau_a)(1+(L-1)(\rho+\theta)-\rho)}\frac{e^{-u}}{u}du \\
&+e^{\tau_a-\tau_s(1+L(\rho+\theta)-\rho)}\int_{u=(\tau_s-\tau_a)(1+\rho+\theta)}^{(\tau_s-\tau_a)(1+(L-1)(\rho+\theta)-\rho)}\frac{e^{u}}{u}du\Bigg) \\
&-\frac{1}{2+L(\rho+\theta)+\theta-\rho}\Bigg(e^{\tau_s-\tau_a(1+L(\rho+\theta)+\theta-\rho)}\int_{u=(\tau_s-\tau_a)(1+\theta)}^{(\tau_s-\tau_a)(1+(L-1)(\rho+\theta)-\rho)}\frac{e^{-u}}{u}du \\
&+e^{\tau_a-\tau_s(1+L(\rho+\theta)+\theta-\rho)}\int_{u=(\tau_s-\tau_a)(1+2\theta+\rho)}^{(\tau_s-\tau_a)(1+L(\rho+\theta)-\rho)}\frac{e^{u}}{u}du\Bigg)\Bigg)\Bigg) \\
&\approx \frac{f(1-f)(1-e^{-\tau_a\rho})}{\rho+\theta}\Bigg(\frac{e^{-\tau_a(L(\rho+\theta)-\rho)}}{2+L(\rho+\theta)-\rho}\Bigg(\\
&e^{-(\tau_s-\tau_a)\theta}\log\left(1+\frac{1}{(\tau_s-\tau_a)(1+\theta)}\right) \\
&-\frac{1}{2}e^{-(\tau_s-\tau_a)((L-1)(\rho+\theta)-\rho)}\log\left(1+\frac{2}{(\tau_s-\tau_a)(1+(L-1)(\rho+\theta)-\rho)}\right) \\
&+e^{-(\tau_s-\tau_a)\theta}\log\left(1+\frac{1}{(\tau_s-\tau_a)(1+(L-1)(\rho+\theta))}\right) \\
&-\frac{1}{2}e^{-(\tau_s-\tau_a)((L-1)(\rho+\theta)-\rho)}\log\left(1+\frac{2}{(\tau_s-\tau_a)(1+\theta+\rho)}\right)\Bigg) \\
&-\frac{e^{-\tau_a(L(\rho+\theta)+\theta-\rho)}}{2+L(\rho+\theta)+\theta-\rho}\Bigg(e^{-(\tau_s-\tau_a)\theta}\log\left(1+\frac{1}{(\tau_s-\tau_a)(1+\theta)}\right) \\
&-\frac{1}{2}e^{-(\tau_s-\tau_a)((L-1)(\rho+\theta)-\rho)}\log\left(1+\frac{2}{(\tau_s-\tau_a)(1+(L-1)(\rho+\theta)-\rho)}\right) \\
&+e^{-(\tau_s-\tau_a)\theta}\log\left(1+\frac{1}{(\tau_s-\tau_a)(1+L(\rho+\theta)-\rho)}\right) \\
&-\frac{1}{2}e^{-(\tau_s-\tau_a)((L-1)(\rho+\theta)-\rho)}\log\left(1+\frac{2}{(\tau_s-\tau_a)(1+2\theta+\rho)}\right)\Bigg)\Bigg).
\end{aligned}
$$

2 Simulated admixture histories

To generate each colored curve in Figure 2 of the main text, we used MS to simulate 4.8×10^{10} bases of pairwise sequence alignment assuming a mutation rate of 2.5×10^{-8} per site per

generation and a recombination rate of 1.0×10^{-8} per site per generation. Letting `ta` denote the admixture time in units of $2N$ generations, the data were generated with the following command line:

```
./ms 2 4800 -t 10000 -r 4000 100000 -I 2 1 1 -es ta/2 1 0.95
-ej ta/2+0.000001 3 2 -ej 0.05 2 1
```

For each of two admixture times $\tau_a = 200$ generations and $\tau_a = 400$ generations, we simulated 100 replicate datasets and inferred the set of demographic parameters τ_a, τ_s, f, and N. The mean and variance of the estimates for each parameter are reported in Table 1 of the main text. Figures S1 and S2 plot the full histogram of values estimated for each of the four parameters, suggesting that all parameters are estimated consistently.

3 Analysis of human data

3.1 Generating empirical tract spectra

We generated empirical spectra of IBS tract lengths from the 1000 Genomes pilot sequences reported in [?] and available at:

`ftp://ftp-trace.ncbi.nih.gov/1000genomes/ftp/pilot_data/release/2010_07/`

We used VCF-tools to extract the haplotype sequences from the VCF files encoding the low-coverage haplotypes and trio haplotypes [?]. We then used the CEU, CHBJPT, and YRI mask files available at

`ftp://ftp.1000genomes.ebi.ac.uk/vol1/ftp/pilot_data/release/2010_03/pilot1/supporting/README_callability_masks`

to excise all haplotype regions that were at least 10,000 bases long and annotated as inaccessible for SNP calling in any of the three low coverage data sets. In addition, we excised all regions annotated as gaps on the UCSC genome browser, a list available at:

`http://cistrome.dfci.harvard.edu/browser/cgi-bin/hgTables`

After these annotated gaps were removed, the remaining genome contained some conspicuously long regions with few or no SNP calls in the low coverage data, meaning a large fraction of the 358 total haplotypes were IBS. By visual inspection on the 1000 genomes browser, many of these SNP deserts had disappeared with the addition of new individuals sequenced after completion of the pilot phase, indicating that their sparsity of SNP calls was probably a sequencing artifact. We therefore excised each 10^6-base region of the genome that did not have at least 66 SNP calls in each of the CEU, YRI, and JPTCHB low coverage data sets.

For the remaining portion of the genome, we generated within-population IBS length spectra as follows: For each low coverage population, we numbered the 120 haplotypes with consecutive integers and aligned haplotype n with haplotype $n+1$. This generated a total of 119 whole-genome alignments, totaling 3.05×10^{11} base pairs, which was cut up into IBS fragments at each of the sites where the two haplotypes differed. For each pair of populations, we also generated a between-population IBS length spectrum by aligning haplotype n from population A with haplotype n from population B, yielding 120 whole-genome alignments that totaled 3.08×10^{11} base pairs. Each alignment was parsed into an IBS length spectrum by cutting it up at the sites where the two haplotypes differed and sorting the resulting fragments by length.

The four parental haplotypes from each trio were numbered 1,2,3,4, and all six pairwise alignments (1 paired with 2, 1 paired with 3, etc.) were used to create the within-population tract spectra. All twelve possible pairwise alignments were used to create the spectrum of CEU-YRI trio sharing.

3.2 IBS tracts in low coverage data

For the CEU and YRI populations, we looked at IBS tract sharing within two subsets of the 1000 Genomes pilot data: four high quality whole genome haplotypes from the trio parents and 120 whole-genome haplotypes sequenced at low coverage. We were able to account for the excess of long IBS tracts in the high coverage trios by modeling the distribution of excess errors in the low coverage data (see Figure 5 of the main text).

One difference between the trios and the low coverage sequences was that the low coverage alignments had higher mean heterozygosity. We found that the YRI low coverage data had a mean heterozygosity of 8.47×10^{-4}, while the YRI trio parents had a mean heterozygosity of 6.98×10^{-4}. To determine whether the difference was significant, we bootstrapped the low coverage data as follows: for $0 \leq n \leq 30$, we subsampled haplotypes $4n, 4n+1, 4n+2$, and $4n+3$ from the low coverage YRI data and determined their shared IBS tract spectrum in the same way that was done with the four trio haplotypes. These bootstrapped low coverage data sets had mean heterozygosity 8.23×10^{-4} with standard deviation 1.81×10^{-5}, making the trio heterozygosity significantly lower. When we bootstrapped the CEU sequences in the same way, the subsample heterozygosities had mean 6.84×10^{-4} and standard deviation 2.3×10^{-5}. In contract, the CEU trio parents had mean heterozygosity 5.50×10^{-4}. In both populations, the low coverage data had an excess heterozygosity between 1.25×10^{-4} and 1.35×10^{-4}, probably due to sequencing errors. The mean heterozygosity between CEU and YRI was also higher in the low coverage data, at 9.33×10^{-4} compared to 8.05×10^{-4} in the trio data.

An error rate of 10^{-4} per base pair would destroy most 10^5- and 10^6-base IBS tracts if the errors were evenly Poisson-distributed throughout the low coverage sequences. However, the situation is somewhat better because of the imputation that was used to generate the

low coverage 1000 Genomes sequences, preferentially calling haplotypes that are IBS with one another in regions where both appear IBD with one of the HapMap references. For this reason, as well as the empirical abundance of long IBS tracts in the low coverage data, we expect true IBS tracts in the low coverage alignments to be broken up by errors at some rate $\epsilon_{\text{IBS}} \ll 10^{-4}$.

Let $f_{\text{trio}}^{\text{YRI}}(L)$ (resp. $f_{\text{lc}}^{\text{YRI}}$) be the frequency of differences between two high coverage YRI samples (resp. two low coverage YRI samples) that are followed by exactly L bases of IBS. If IBS tracts are accurately observed in the trio data but broken up by errors at rate $\epsilon_{\text{IBS}}^{\text{YRI}}$ in the low coverage data, then we should expect that $f_{\text{lc}}^{\text{YRI}}(L) \approx f_{\text{trio}}^{\text{YRI}}(L)e^{-L\epsilon_{\text{IBS}}^{\text{YRI}}}$. In this way, the data are consistent with an error rate of $\epsilon_{\text{IBS}}^{\text{YRI}} = 5 \times 10^{-6}$, with the function $f_{\text{trio}}^{\text{YRI}}(L)e^{-L\epsilon_{\text{IBS}}^{\text{YRI}}}$ falling within the realm of variation of $f_{\text{lc}}^{\text{YRI}}(L)$ in the bootstrapped low coverage datasets. The frequencies of long IBS tracts in the trio data are consistent with an identical error rate of $\epsilon_{\text{IBS}}^{\text{CEU}} = 5 \times 10^{-6}$ (see Figure S4).

3.3 Human evolutionary model

The simulated human data plotted in Figure 8 of the main text were generated using the following MS command line:

```
./ms 2 1800 -t 10000 -r 4000 100000 -I 2 2 2 -en 0 2 N0_YRI
-en t0_YRI 2 N1_YRI -en t_med 2 N2 -eN t_ancient N3 -en 0 1 N0_CEU
-en t0_CEU 1 N1_CEU  -en t_s 1 N2 -es t_m 1 (1-f_{Eu-As}/4)
-es t_m 2 (1-f_{Eu-As}/4) -ej t_m*1.00001 3 2 -ej t_m*1.00001 4 1
-es t_m 1 1-f_{Eu-As}/4 -es t_m+(t_s-t_m)/4 2 1-f_{Eu-As}/4
-ej (t_m+(t_s-t_m)/4)*1.00001 5 2 -ej (t_m+(t_s-t_m)/4)*1.00001 6 1
-es (t_m+(t_s-t_m)/2) 1 (1-f_{Eu-As}/4) -es (1-f_{Eu-As}/4) 2 (1-f_{Eu-As}/4)
-ej (t_m+(t_s-t_m)/2)*1.00001 7 2 -ej (t_m+(t_s-t_m)/2)*1.00001 8 1
-es (t_m+(t_s-t_m)*3/4) 1 (1-f_{Eu-As}/4)
-es (t_m+(t_s-t_m)*3/4) 2 (1-f_{Eu-As}/4) -ej (t_m+(t_s-t_m)*3/4)*1.00001 9 2
-ej (t_m+(t_s-t_m)*3/4)*1.00001 10 1-es t_s 1 1-f_{ghost}
-ej t_s*1.00001 11 2 -ej t_ghost 1 2
```

The migration rate $m_{\text{Eu-As}}$ in Table 2 of the main text is calculated such that $f_{\text{Eu-As}} = m_{\text{Eu-As}}(t_s - t_m)$, letting continuous migration be approximated by four evenly spaced discrete pulses.

To estimate parameters efficiently, we used a two-step procedure. First, we estimated N_2, N_3, and t_{ancient} by fitting a simple bottleneck history to the IBS sharing with the YRI. Next, we fixed N_2, N_3, and t_{ancient} and estimated the rest of the parameters by jointly maximizing the likelihood of all three informative spectra: within the YRI, within the CEU and between YRI and CEU.

3.4 Assessing uncertainty via simulation

We simulated 30 replicate datasets under the human evolutionary model described in section **??** with the maximum likelihood parameters inferred from the trio data. We then estimated parameters from each replicate dataset, using parametric bootstrapping to gauge our accuracy at inferring a complex history. Figure S10 illustrates the differences between the parameters inferred from the trio data and the mean estimates obtained from replicate simulations. Figures S7, S8 and S9 record the full distribution of parameter estimates obtained from simulated data.

4 Comparison to other demographic methods

4.1 Using site frequencies vs. IBS tracts to infer simple admixture histories

Like our method, the program ∂a∂i by Gutenkunst, *et al.* can compute a composite likelihood of genomic data given a wide range of parametric histories [**?**]. We therefore evaluated ∂a∂i's ability to infer the parameters of the simple admixture history considered in the main text.

In Figure 3, we compare composite likelihood surfaces generated by ∂a∂i from the joint allele frequency spectrum and by our method from the joint IBS tract spectrum. Each point in the ∂a∂i likelihood surface is generated by fixing τ_a and τ_s and then optimizing f (∂a∂i deterministically optimizes the size N). Similarly, each point in the IBS tract likelihood surface is generated by fixing τ_a and τ_s and jointly optimizing f and N. These likelihood surfaces show that both methods allow for accurate grid search estimation of demographic parameters. However, the ∂a∂i numerical optimization routines usually fail to arrive at a good estimate starting from a random point in demographic parameter space. For the $\tau_a = 0.01$ history, the optimal parameters located by grid search have a Poisson log likelihood greater than $-5,000$, the best parameters obtained from 20 random Nelder-Mead optimizations have Poisson log likelihood $-8,329$. Nelder-Mead optimization was chosen for this comparison because it is the routine recommended in the ∂a∂i manual for optimization starting far from the true optimum. In contrast, if we sample Θ uniformly at random from a bounded range and maximize the likelihood of observed IBS tracts, the optimization consistently terminates very close to the global maximum (see Supplementary Table 1). Both the SFS likelihood surface and the IBS tract likelihood surface allow for parameter estimation by grid search, but the two likelihood surfaces have different shapes that suggest complementary demographic sensitivities. The SFS likelihood is more sensitive to variation in divergence time than to changes in admixture time, while the IBS tract likelihood is more sensitive to variation in the time of last gene flow.

4.2 The NIEHS site frequency spectrum

Although the Gutenkunst, *et al.* and Gravel, *et al.* histories fit human site frequency spectra well, Figure 5 illustrates that they do not predict the right spectrum of shared IBS tracts. Similarly, there is no guarantee that our inferred demographic history should predict the right CEU-YRI site frequency spectrum. To test this, we used MS to simulate a 20-by-20 site frequency spectrum under our demographic model and compared it to the National Institute of Environmental Health Science (NIEHS) frequency spectrum that was analyzed in [**?**] and generously made available by Ryan Gutenkunst. The simulated SFS had an FST of 0.205, significantly larger than the FST value of 0.158 that was computed from the NIEHS frequency spectrum data (see Figure S11). We discuss possible reasons for the discrepancy in the main text.
